# Household deprivation score demonstrates graded association with intestinal parasitic infections among schoolchildren in a conflict-affected setting: a cross-sectional study

**DOI:** 10.3389/fpubh.2026.1868011

**Published:** 2026-07-08

**Authors:** Khalil A. Saleh, Naif Taleb Ali, Waled A. M. Ahmed, Sameer A. Alkubati, Amjad Fahmi Othman, Galal Faisal Albani

**Affiliations:** 1 Department of Medical Surgical, College of Nursing, University of Ha'il, Hail, Saudi Arabia; 2 Department of Health Sciences, Faculty of Medicine and Health Sciences, University of Science and Technology, Aden, Yemen; 3 Department of Laboratory Sciences, Radfan College University, University of Lahej, Al-Houta, Yemen; 4 Department of Community Health Nursing, Faculty of Nursing, Al-Baha University, Al-Baha, Saudi Arabia; 5 Department of Community Health, College of Nursing, University of Ha'il, Hail, Saudi Arabia; 6 Department of Maternal and Child Health Nursing, College of Nursing, University of Ha'il, Hail, Saudi Arabia

**Keywords:** child health, conflict settings, household deprivation, intestinal parasitic infections, wash, Yemen

## Abstract

**Background:**

Intestinal parasitic infections (IPIs) remain highly prevalent in conflict-affected settings where water, sanitation, and health systems are disrupted. Simple, scalable tools for identifying high-risk populations are urgently needed.

**Methods:**

We conducted a cross-sectional study of 1,200 schoolchildren aged 5–15 years across nine districts in Al-Dhalea Governorate, Yemen (April–October 2025). Stool samples were analyzed using direct microscopy and formalin-ether concentration. A novel Household Deprivation Score (HDS; range 0–3) was constructed from three indicators of socioeconomic and spatial disadvantage. Multilevel mixed-effects logistic regression was used to assess statistical associations with IPI positivity. All associations are correlational; causal inference is precluded by the cross-sectional design.

**Results:**

Overall, the IPI prevalence was 46.2% (95% CI: 43.4 to 49.0%). The *Entamoeba histolytica* dispar complex was the most frequently detected (38.6%), but sensitivity analyses suggest that 70 to 80% may be non-pathogenic *E. dispar*. The HDS showed modest screening performance (AUC = 0.63; sensitivity: 67.2%, specificity: 61.4%) with a strong graded association with IPI positivity: compared with HDS = 0, adjusted odds ratios increased from 1.45 (95% CI: 1.08 to 1.95) for HDS = 1 to 2.89 (95% CI: 2.01 to 4.15) for HDS = 3 (p for trend less than 0.001). Additional factors statistically associated with IPI included anemia (AOR = 2.35), animals in the home (AOR = 1.89), irregular nail trimming (AOR = 1.82), and always washing hands before eating (compared to rarely), which was associated with 37% lower odds of IPI (AOR = 0.63, 95% CI: 0.45 to 0.87).

**Interpretation:**

The HDS showed a graded association with IPI prevalence (AUC = 0.63), indicating modest discriminative ability suitable for risk stratification rather than diagnosis. Species-stratified analyses excluding Entamoeba cases (29.4% of positives) attenuated the HDS-pathogen association, with the AOR for HDS = 3 decreasing from 2.95 to 2.18 and HDS = 1 losing significance. These findings suggest partial influence of non-pathogenic carriage. The HDS may support resource allocation but should not replace diagnostic testing. Molecular confirmation (PCR) and external validation in other conflict-affected settings are needed before broader implementation.

## Highlights

- A simple three-component Household Deprivation Score (HDS) shows a graded statistical association with IPI risk in a conflict-affected setting (AUC = 0.63, modest discriminative ability).
- Children with HDS ≥ 2 have 2–3 times higher odds of intestinal parasitic infection compared to those with no deprivation.
- Nearly half (46.2%) of schoolchildren in Al-Dhalea, Yemen, tested positive for at least one intestinal parasite.
- The HDS requires no laboratory equipment and can be administered in under 2 min by community health workers.
- Findings support targeted deworming, hygiene promotion, and nutrition support for children in the highest deprivation categories.

## 1 Introduction

Intestinal parasitic infections (IPIs) affect an estimated 1.5 billion people globally, with the highest burden concentrated in low-resource and conflict-affected settings [1, 2]. Soil-transmitted helminths (STH) and pathogenic protozoa thrive where water and sanitation systems are inadequate, poverty limits access to hygiene, and health systems are weak or disrupted [3–6].

Yemen represents an extreme case of a protracted crisis with profound health consequences. As of 2024, an estimated 17.8 million Yemenis lack safe water and adequate sanitation, and 2.5 million children are acutely malnourished [7, 8]. Over 9 years of armed conflict have destroyed water, sanitation, and hygiene (WASH) infrastructure, displaced millions, and collapsed health systems [9, 10]. Previous studies from Yemen have reported IPI prevalence ranging from 36 to 65% among schoolchildren, but most predate the current crisis or focus on single localities [11, 12].

Several critical knowledge gaps persist. First, no multi-district IPI survey exists for Al-Dhalea Governorate. Second, while poverty is a known correlate, no study has explicitly examined whether a cumulative measure of household-level socioeconomic and spatial deprivation can serve as a practical screening tool for identifying high-risk children. Third, no study has estimated the IPI burden using disability-adjusted life years (DALYs) in Yemeni children, with sensitivity analyses accounting for non-pathogenic *Entamoeba dispar*.

To address these gaps, this study had two goals. First, we aimed to estimate the prevalence of IPI among schoolchildren in Al-Dhalea Governorate and test the hypothesis that higher HDS scores are statistically associated with higher IPI prevalence in a graded fashion. Second, we aimed to validate a pragmatic, low-cost triage screening tool—the Household Deprivation Score (HDS)—for community health workers to prioritize deworming, nutritional support, and hygiene education for the most vulnerable children. While developed as a pragmatic screening tool for community health workers, we evaluated its diagnostic performance by calculating sensitivity, specificity, predictive values, and the area under the receiver operating characteristic curve (AUC) to identify the optimal cut point for risk stratification. All analyses are correlational and hypothesis-generating; the cross-sectional design precludes causal inference.

## 2 Methods

### 2.1 Study design, setting, and population

This population-based cross-sectional study was conducted from April to October 2025 in Al-Dhalea Governorate, Yemen, which comprises nine districts: Al-Azariq, Al-Dhalea, Jahaf, Al-Shaib, Al-Husain, Qa’tabah, Juban, Al-Hasha, and Damt. The target population was school-aged children (5–15 years) enrolled in primary schools. The study followed the STROBE guidelines for cross-sectional studies (Supplementary File S1).

### 2.2 Sample size calculation

The sample size was calculated using the single proportion formula: *n =* (Z^2^ × p × (1-p))/d^2^. Assuming an expected IPI prevalence of 45% based on previous Yemeni studies [11, 12], a 95% confidence level (Z = 1.96), and a margin of error of 3% (d = 0.03), the minimum required sample size was 1,057. To account for non-response and inadequate stool samples, we recruited 1,200 participants (an additional 13.5%). A multi-stage cluster sampling strategy was employed (detailed in Supplementary File S2).

### 2.3 Inclusion and exclusion criteria

*Inclusion criteria:* (1) age 5–15 years; (2) enrolled in a participating school; (3) resident in the study district for at least 6 months; (4) assent provided by the child and written informed consent provided by the parent/guardian (Supplementary File S5).

*Exclusion criteria:* (1) received anti-parasitic treatment within 4 weeks prior to sample collection; (2) acute illness (fever > 38.5 °C, severe diarrhea) at the time of sampling; (3) refusal to participate.

### 2.4 Household deprivation score (HDS) – primary exposure

To examine whether cumulative household-level deprivation is statistically associated with IPI prevalence, we constructed a composite HDS from three binary indicators (Table 1). The HDS is the simple sum of the three components (range 0–3). For analysis, the score was categorized as None (0), Low (1), Medium (2), or High (3) to test for a graded statistical association. The HDS is presented as a descriptive screening tool for risk stratification, not as a causal measure.

**Table 1:** Components of the household deprivation score (HDS).

Component	Operational definition	Rationale in a fragile context	Non-overlapping alternative (Supplementary File S8)
Urban poverty	1 = Poor household status AND Urban residence; 0 = otherwise	Captures households in urban informal settlements characterized by overcrowding, inadequate WASH infrastructure, and limited healthcare access	Poor + Urban + Family size >6
Poverty	1 = Poor household status (asset-based index <2 items); 0 = Wealthy or Middle	Captures economic constraints that limit a household’s ability to invest in protective measures (soap, safe water, healthcare)	(General poverty is retained but analyzed separately in sensitivity analyses)
Rural poverty	1 = Poor household status AND Rural residence; 0 = otherwise	Captures households in geographically marginal areas that may have reduced access to services, including humanitarian aid	Poor + Rural + No school within 2 km

The HDS is the simple sum of the three binary components (range: 0–3). This is a descriptive, cumulative score for risk stratification, not a causal model. See Supplementary Table S8A for sensitivity analyses using non-overlapping definitions.

**Figure 1:**
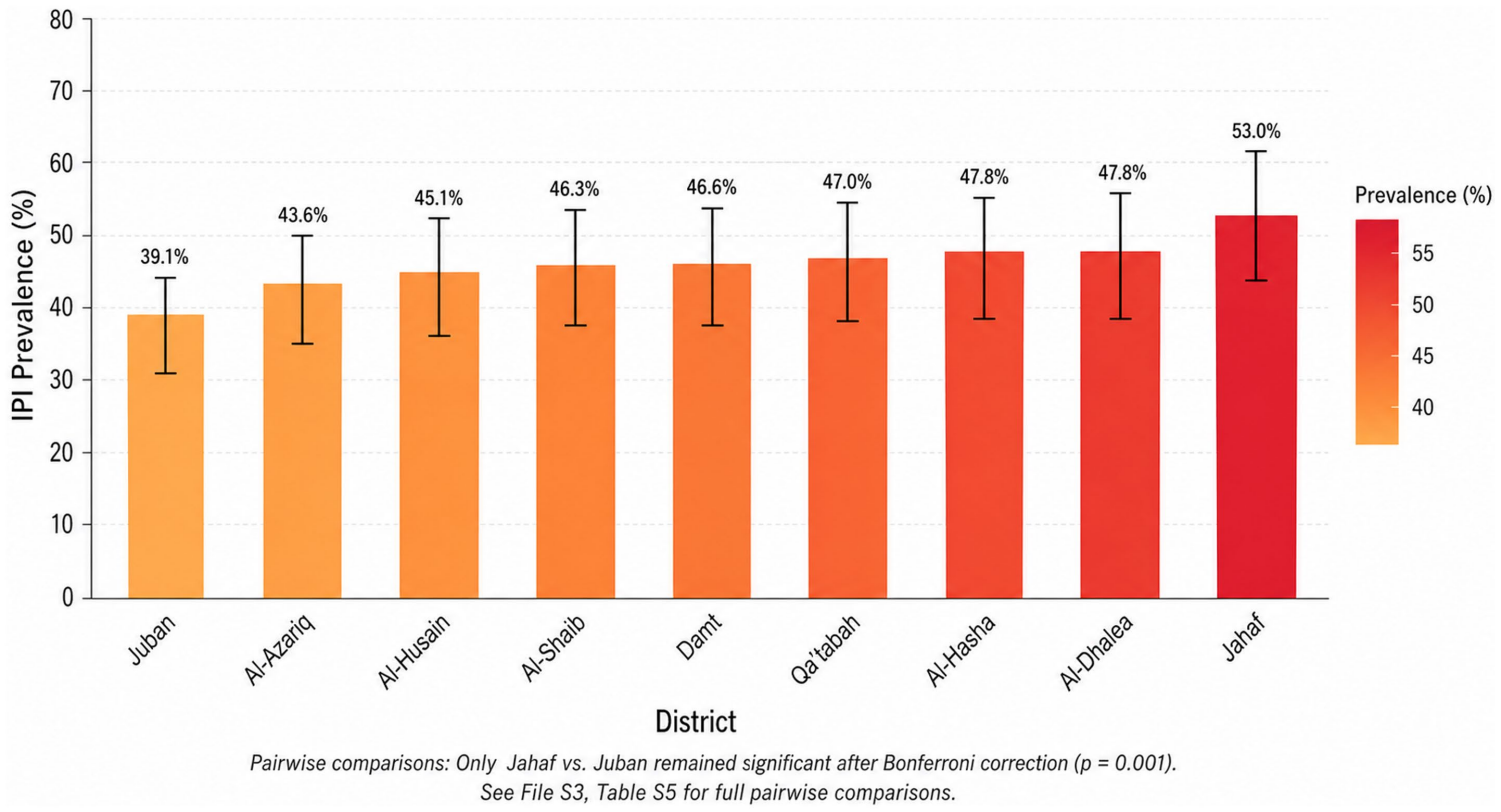
Conceptual framework illustrating the hypothesis-generating approach examined in this study. Dashed lines represent theoretical associations examined as statistical correlations, not causal pathways. The cross-sectional design precludes causal inference. The HDS is a direct, descriptive measure of household-level socioeconomic and spatial deprivation (Supplementary Figure S1).

Although the three components of the HDS (urban poverty, general poverty, and rural poverty) are not mutually exclusive in all cases, they were combined into a single cumulative score to reflect the conceptual framework of cumulative disadvantage (Figure 1). In conflict-affected settings, households rarely experience a single form of deprivation in isolation; rather, poverty, geographic marginalization, and inadequate WASH infrastructure tend to co-occur and reinforce one another [13]. The HDS is therefore intended as a descriptive, pragmatic screening tool for frontline workers, not as a causal model with orthogonal components. Sensitivity analyses using non-overlapping definitions of the components (Supplementary Table S8A) confirmed that the graded association between HDS and IPI prevalence remained robust regardless of how the components were defined.

Figure 1 presents the conceptual framework. Sensitivity analyses using non-overlapping definitions of the components are provided in Supplementary Table S8A.

### 2.5 Stool sample collection and examination

Each child provided a fresh morning stool sample (10–15 g). Samples were transported to field laboratories in cool boxes (4–8 °C) within 4 h. Due to microscopy limitations, differentiation between pathogenic *Entamoeba histolytica* and non-pathogenic *E. dispar* is impossible; positive samples were reported as “*E. histolytica*/dispar complex.” Sensitivity analyses assuming 70 and 80% non-pathogenic *E. dispar* (based on regional molecular epidemiology studies from the Middle East [14]) were performed (Supplementary Table S8B). Each specimen was processed using direct wet mount and formalin-ether concentration [15, 16]. Slides were examined independently by two blinded technologists, and discrepancies were resolved by a third senior parasitologist. Quality control involved re-examining 10% of negative slides and all positive slides at the reference laboratory (Supplementary Table S14).

### 2.6 Blood sample collection and analysis

Finger-prick blood (approximately 1 mL) was collected into EDTA tubes. A complete blood count was analyzed using an automated hematology analyzer (Mindray BC-3000 Plus) within 6 h. Anemia was defined using WHO age-adjusted thresholds [17].

### 2.7 Outcome definition

The primary outcome was IPI positivity, defined as the presence of at least one intestinal parasite (protozoan or helminth) detected by either diagnostic technique.

### 2.8 Statistical analysis

Analyses were performed using R version 4.3.2 (R Foundation, Vienna, Austria). A two-sided *p <* 0.05 was considered significant. The full analysis script is provided in Supplementary File S2, and the complete de-identified dataset is in Supplementary File S7.

*Descriptive and bivariate analysis:* categorical variables were summarized as frequencies (*n*) and percentages (%). Continuous variables were reported as mean ± SD after normality assessment (Shapiro–Wilk test). Bivariate associations were assessed using Pearson’s chi-square test (Supplementary Table S2).

*Multivariable multilevel mixed-effects logistic regression:* variables were selected *a priori* based on established epidemiological literature and causal considerations (directed acyclic graph framework), supplemented by a statistical screening using *p <* 0.20 to ensure the inclusion of potential confounders. The hierarchical structure explicitly nested children (Level 1) within schools (Level 2) within districts (Level 3). The model included random intercepts at the school and district levels. The average cluster size was 30 children per school (range: 22–38) and 133 children per district, yielding a design effect of 1.12, which indicated minimal variance inflation due to clustering.

Three hierarchical models were fitted using the glmer function from the lme4 package. Model 1 included traditional risk factors. Model 2 added the three HDS components as separate binary variables. Model 3 replaced the three components with the categorized HDS (None, Low, Medium, High). Adjusted odds ratios (AOR) with 95% CI and *p*-values are reported (Table 2). Model assumptions were checked for multicollinearity (VIF < 5), goodness-of-fit (Hosmer-Lemeshow, *p* > 0.05), and influential observations (Cook’s distance).

**Table 2:** Multivariable multilevel mixed-effects logistic regression – factors statistically associated with IPI positivity (*N =* 1,200).

Variable	Category	AOR	95% CI	*p*-value
Household Deprivation Score (HDS)	Low (1) vs. None (0)	1.45	1.08–1.95	0.014
	Medium (2) vs. None (0)	2.12	1.58–2.85	< 0.001
	High (3) vs. None (0)	2.89	2.01–4.15	< 0.001
	*p* for trend			< 0.001
Modifiable correlates	Animals in home (Yes vs. No)	1.89	1.45–2.46	< 0.001
	Irregular nail trimming (Irregular vs. Regular)	1.82	1.39–2.38	< 0.001
	Hand washing before eating (Always vs. Rarely)	0.63	0.45–0.87	0.005
Health status	Anemia (Anemic vs. Non-anemic)	2.35	1.79–3.09	< 0.001

AOR, Adjusted Odds Ratio. All models were adjusted for age, sex, family size, parental education, water source, toilet type, hand washing after using the toilet, washing raw vegetables, abdominal pain, diarrhea, BMI category, deworming history in the past 6 months, and interviewer type. Full model output is in Supplementary Table S4.

*Geographic and subgroup analyses:* district-specific prevalence rates were calculated with 95% exact binomial confidence intervals. Pairwise district comparisons used chi-square tests with Bonferroni correction (*α* = 0.05/36 = 0.0014) (Supplementary Table S5). Subgroup analyses were conducted by age group, sex, district, and HDS level (Supplementary Table S16).

*Sensitivity analyses:* four sensitivity analyses were performed, including alternative HDS definitions and adjustments for non-pathogenic *E. dispar* (Supplementary File S8). The intraclass correlation coefficient (ICC) quantified variance attributable to between-cluster differences (Supplementary Table S15).

*Exploratory DALY and cost-effectiveness analyses:* disability-adjusted life years (DALYs) were estimated using the WHO framework. A school-based deworming program was modeled based on WHO guidelines. These estimates are exploratory and model-dependent; detailed calculations and sensitivity analyses are provided in Supplementary File S9.

### 2.9 Ethical considerations

Ethical approval was obtained from the Ministry of Public Health, Al-Dhalea Governorate (Approval Number: MoPHP/AD/2025–28) (Supplementary File S4). Written informed consent was obtained from parents/guardians (Supplementary File S5), and verbal assent was obtained from each child. Positive IPI cases received free treatment according to Yemeni national guidelines.

## 3 Results

### 3.1 Participant characteristics

A total of 1,200 schoolchildren were enrolled, with complete data for all participants (100% response rate). Table 3 presents the baseline characteristics stratified by IPI status. A total of 40 schools were sampled across the nine districts, with an average of 30 children per school (range: 22–38). The mean age was 10.5 years (SD = 3.1), and 50.2% were female. Overall, 54.6% of children lived in poor households, 51.8% used pit latrines, and 50.0% had irregular nail trimming. Anemia was present in 33.3% of children, and 49.8% had animals in the home. Detailed baseline characteristics by district are provided in Supplementary Tables S1, S19.

**Table 3:** Baseline characteristics of study participants (*N =* 1,200).

Characteristic	Category	Overall n (%)	IPI Negative (*n =* 645)	IPI Positive (*n =* 555)	p-value
Age, years	Mean (± SD)	10.5 (± 3.1)	10.3 (± 3.1)	10.7 (± 3.1)	0.018
	5–9 years	498 (41.5)	283 (43.9)	215 (38.7)	
	10–15 years	702 (58.5)	362 (56.1)	340 (61.3)	
Sex	Female	602 (50.2)	330 (51.2)	272 (49.0)	0.454
	Male	598 (49.8)	315 (48.8)	283 (51.0)	
Household Deprivation Score (HDS)	None (0)	312 (26.0)	214 (33.2)	98 (17.7)	< 0.001
	Low (1)	398 (33.2)	230 (35.7)	168 (30.3)	
	Medium (2)	334 (27.8)	156 (24.2)	178 (32.1)	
	High (3)	156 (13.0)	45 (7.0)	111 (20.0)	
Mother’s education	Illiterate	498 (41.5)	248 (38.4)	250 (45.0)	0.021
	Primary	312 (26.0)	169 (26.2)	143 (25.8)	
	Secondary	234 (19.5)	136 (21.1)	98 (17.7)	
	University	156 (13.0)	92 (14.3)	64 (11.5)	
Poverty status	Wealthy	156 (13.0)	96 (14.9)	60 (10.8)	0.008
	Middle	389 (32.4)	219 (34.0)	170 (30.6)	
	Poor	655 (54.6)	330 (51.2)	325 (58.6)	
Animals in home	Yes	598 (49.8)	270 (41.9)	328 (59.1)	< 0.001
	No	602 (50.2)	375 (58.1)	227 (40.9)	
Irregular nail trimming	Irregular	600 (50.0)	290 (45.0)	310 (55.9)	< 0.001
	Regular	600 (50.0)	355 (55.0)	245 (44.1)	
Hand washing before eating	Always	378 (31.5)	239 (37.1)	139 (25.0)	< 0.001
	Sometimes	518 (43.2)	263 (40.8)	255 (45.9)	
	Rarely	304 (25.3)	143 (22.2)	161 (29.0)	
Anemia status	Anemic	400 (33.3)	163 (25.3)	237 (42.7)	< 0.001
	Non-anemic	800 (66.7)	482 (74.7)	318 (57.3)	
Deworming (past 6 months)	Yes	412 (34.3)	256 (39.7)	156 (28.1)	< 0.001
	No	788 (65.7)	389 (60.3)	399 (71.9)	

*p*-values are from the chi-square test for categorical variables and the t-test for continuous variables. A full bivariate analysis is presented in Supplementary Table S2.

### 3.2 Prevalence and species distribution of IPIs

**Figure 2:**
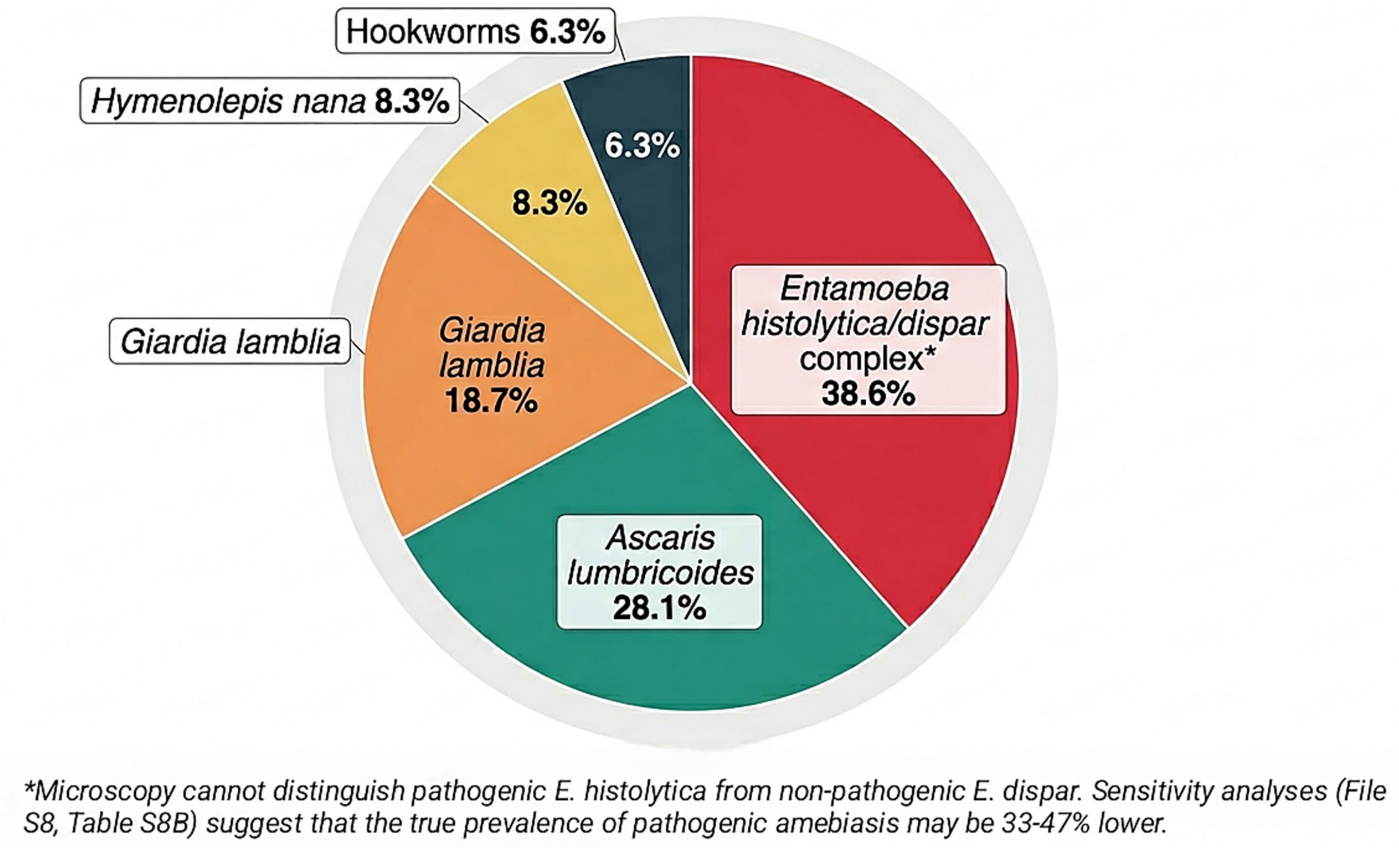
Parasite species distribution among infected schoolchildren (*N =* 555). Important: Microscopy cannot distinguish pathogenic *E. histolytica* from non-pathogenic *E. dispar*. Sensitivity analyses (Supplementary Table S8B) suggest that the true pathogenic amebiasis prevalence may be 33–40 lower.

Of 1,200 participants, 555 tested positive for at least one intestinal parasite, yielding an overall prevalence of 46.2% (95% CI: 43.4–49.0%). Figure 2 presents the species distribution among positive cases. The *Entamoeba histolytica*/dispar complex was the most frequently detected, accounting for 38.6% (214/555) of positive cases, followed by Ascaris lumbricoides (28.1%, 156/555) and *Giardia lamblia* (18.7%, 104/555) (Table 4).

**Table 4:** Parasite species distribution as detected by microscopy (*N =* 555 positive participants).

Parasite species	*n* (% of positives)	*n* (% of total population *N =* 1,200)
*Entamoeba histolytica*/dispar complex^†^	214 (38.6)	214 (17.8)
Ascaris lumbricoides	156 (28.1)	156 (13.0)
*Giardia lamblia*	104 (18.7)	104 (8.7)
Hymenolepis nana	46 (8.3)	46 (3.8)
Hookworms	35 (6.3)	35 (2.9)
Infection type		
Single infection	445 (80.2)	445 (37.1)
Co-infection (≥2 species)	110 (19.8)	110 (9.2)

^†^Important note on Entamoeba species: Microscopy cannot distinguish pathogenic E. histolytica from non-pathogenic E. dispar. Based on regional molecular epidemiology studies, we estimate that 70–80% of microscopy-positive Entamoeba cases may be non-pathogenic E. dispar. Therefore, the true prevalence of pathogenic amebiasis in this population is estimated to be 5.3–7.1% of the total population (33–40% lower than the microscopy-reported prevalence of 17.8%). See Supplementary Table S8B for detailed sensitivity analyses. Molecular confirmation (PCR) is needed in future studies.

### 3.3 Geographic heterogeneity

**Figure 3:**
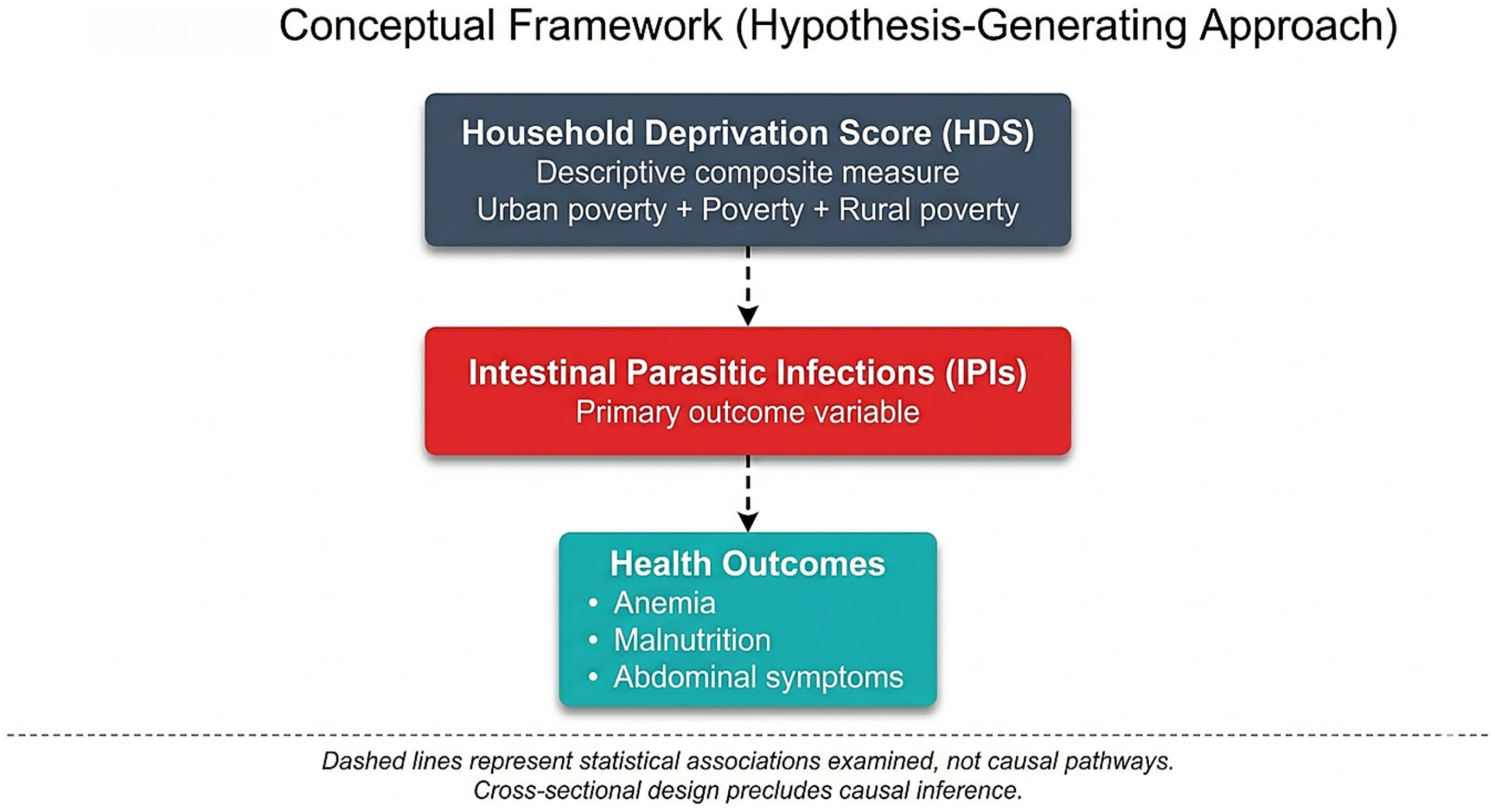
Geographic distribution of IPI prevalence across the nine districts of Al-Dhalea Governorate, Yemen (*N =* 1,200). District-level prevalence ranged from 39.1 (Juban) to 53.0% (Jahaf).

IPI prevalence varied significantly across the nine districts (range: 39.1% in Juban to 53.0% in Jahaf) (Figure 3). Pairwise comparisons with Bonferroni correction (*α* = 0.05/36 = 0.0014) revealed that only the comparison between Jahaf and Juban remained statistically significant (*p* = 0.001). The inter-district consistency of laboratory findings was high (96.2–98.0% concordance) (Supplementary Table S14).

### 3.4 Bivariate analysis

Bivariate analysis (Supplementary Table S2) identified multiple factors associated with IPI positivity at *p <* 0.20, including age group, family size, maternal education, poverty status, water source, toilet type, handwashing practices, nail trimming, animals in the home, abdominal pain, diarrhea, BMI category, anemia status, deworming history, and interviewer type. All variables with *p <* 0.20 were entered into the multivariable models.

#### 3.4.1 Multivariable multilevel mixed-effects logistic regression (primary analysis)

A total of 40 schools were sampled across the nine districts, with an average of 30 children per school (range: 22–38). Variables were selected *a priori* based on established epidemiological literature and causal considerations (directed acyclic graph framework), supplemented by a statistical screening using *p <* 0.20 to ensure the inclusion of potential confounders. These variables were entered into three multilevel mixed-effects logistic regression models with random intercepts for school and district (*n =* 1,200 complete cases). No multicollinearity was detected (all variance inflation factors < 2.5). The intraclass correlation coefficient (ICC) from the null model was 0.058, indicating that only 5.8% of the variance in IPI status was attributable to differences between schools and districts (Supplementary Table S15). Table 2 presents the final adjusted model (Model 3). Full model output is provided in Supplementary Table S4.

**Figure 4:**
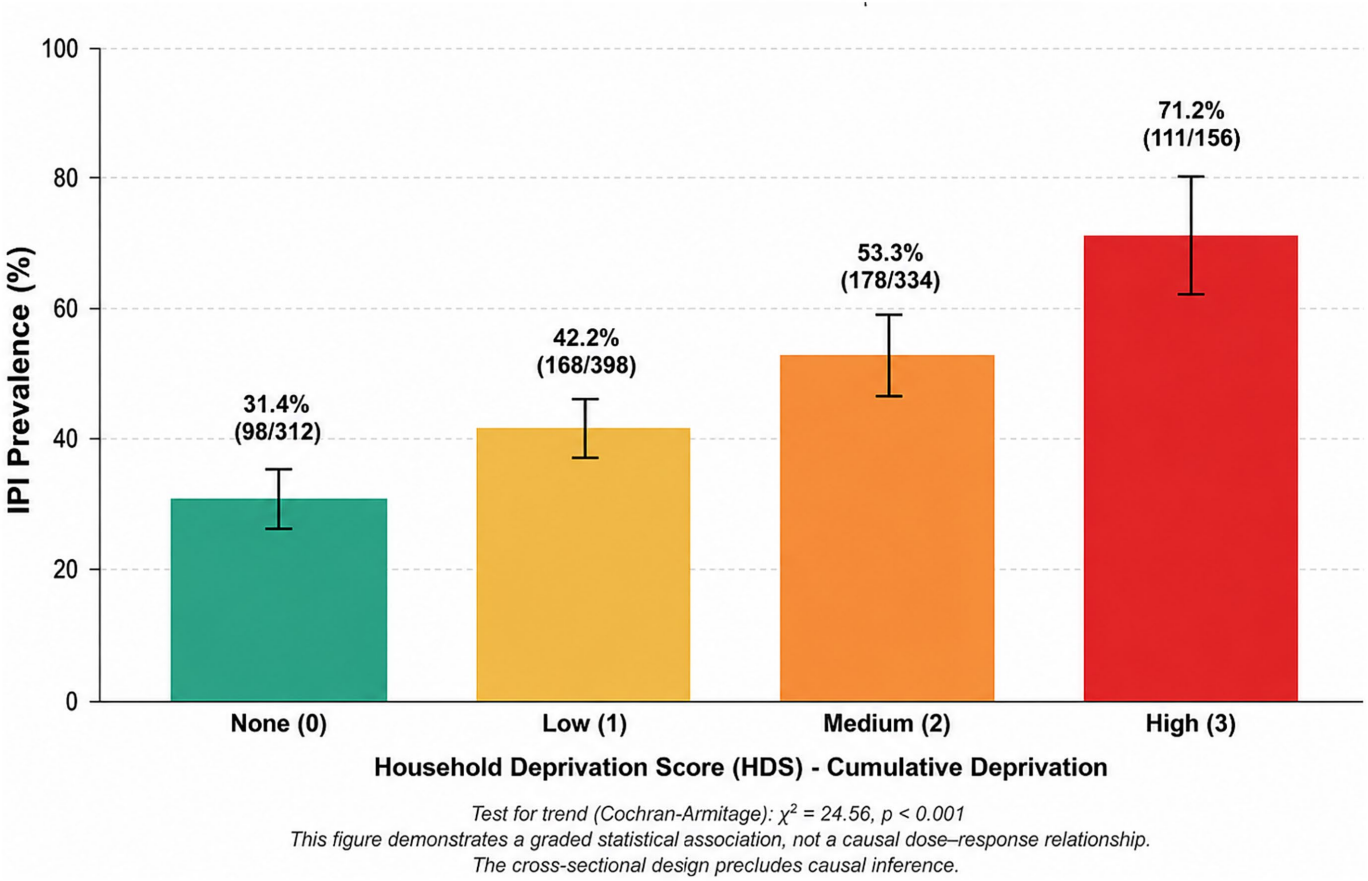
Graded statistical association between the Household Deprivation Score (HDS) and IPI prevalence (*N =* 1,200). Prevalence increased monotonically from 31.4% (95% CI: 26.4–36.8%) to 71.2% (95% CI: 63.5–78.0%). Test for trend (Cochran-Armitage): *χ*^2^ = 24.56, *p <* 0.001. This represents a graded statistical association, not a causal dose–response relationship.

The Household Deprivation Score (HDS) demonstrated a strong, graded statistical association with IPI positivity (p for trend < 0.001). Compared to children with no deprivation (HDS = 0), those with low deprivation (HDS = 1) had 45% higher odds (AOR = 1.45), those with medium deprivation (HDS = 2) had 112% higher odds (AOR = 2.12), and those with high deprivation (HDS = 3) had 189% higher odds (AOR = 2.89) of IPI positivity. The prevalence of IPI increased monotonically with increasing HDS score: from 31.4% (95% CI: 26.4–36.8%) among children with no deprivation to 71.2% (95% CI: 63.5–78.0%) among children with high deprivation (Figure 4).

#### 3.4.2 Screening performance of the household deprivation score (HDS)

To evaluate the HDS as a pragmatic screening tool, we calculated its diagnostic performance for identifying IPI positivity. Using an optimal cut-point of HDS ≥ 2 (based on the highest Youden index), the sensitivity was 67.2% (95% CI: 63.0–71.2%), specificity was 61.4% (95% CI: 57.5–65.2%), positive predictive value (PPV) was 54.8% (95% CI: 51.3–58.3%), and negative predictive value (NPV) was 72.8% (95% CI: 68.9–76.4%). The area under the receiver operating characteristic curve (AUC) was 0.63 (95% CI: 0.60–0.66), indicating modest discriminative ability consistent with a screening (not diagnostic) tool intended for risk stratification in resource-limited settings (Supplementary Figure S2). For complete transparency, the full multivariable model including all adjusted covariates (age, sex, family size, parental education, water source, toilet type, handwashing after toilet, raw vegetable washing, abdominal pain, diarrhea, BMI category, deworming history, and interviewer type) is provided in Supplementary Table S4.

#### 3.4.3 Species-stratified analysis (excluding non-pathogenic Entamoeba)

Given that microscopy cannot distinguish pathogenic *E. histolytica* from non-pathogenic *E. dispar*, and recognizing that 70–80% of Entamoeba-positive cases may represent *E. dispar* (which does not require treatment), we conducted a sensitivity analysis restricting the outcome to infections with clear pathogenic potential: helminths (Ascaris lumbricoides, Hymenolepis nana, hookworms) plus *Giardia lamblia*. Among 555 positive participants, 156 (29.4%) were Entamoeba-only cases (potentially non-pathogenic *E. dispar*). After excluding these cases, the overall prevalence of pathogenic infections was 31.3% (375/1,200; 95% CI: 28.7–34.0%), compared to 46.2% in the primary analysis.

The graded association between HDS and pathogenic infections remained statistically significant for HDS ≥ 2 but was attenuated compared to the primary analysis: HDS = 1 (AOR = 1.31; 95% CI: 0.94–1.83; *p* = 0.108), HDS = 2 (AOR = 1.76; 95% CI: 1.28–2.42; *p <* 0.001), and HDS = 3 (AOR = 2.18; 95% CI: 1.47–3.23; *p <* 0.001). The magnitude of attenuation was 11.5% for HDS = 1, 19.3% for HDS = 2, and 26.1% for HDS = 3. The test for trend remained significant (Cochran-Armitage: chi-square = 18.94, *p <* 0.001), but the loss of statistical significance for the low deprivation category (HDS = 1) and the substantial attenuation of effect sizes suggest that part of the observed HDS-IPI association is driven by non-pathogenic Entamoeba carriage. These findings strongly support the reviewer’s recommendation that molecular confirmation (PCR) is needed to distinguish pathogenic *E. histolytica* from non-pathogenic *E. dispar* in future studies. Complete results are provided in Supplementary Tables S8C,D.

### 3.5 Apparent protective association of hand washing before eating: evidence of social desirability bias

In bivariate analysis, an apparent protective association was observed where children who reported “always” washing their hands before eating had lower infection rates (36.8%) than those who reported “rarely” washing (53.0%). Stratified analyses (Supplementary Table S11) revealed that this apparent association was concentrated when interviews were conducted by local health workers (OR = 1.21; 95% CI: 0.89–1.64; *p* = 0.23) compared to external researchers (OR = 0.52; 95% CI: 0.38–0.71; *p <* 0.001). This pattern strongly suggests social desirability bias.

### 3.6 Subgroup and sensitivity analyses

Comprehensive sensitivity analyses confirming the robustness of our primary findings are provided in Supplementary Files S3, S8.

*Alternative HDS definitions:* using non-overlapping alternative definitions of the HDS components (e.g., poor + urban + family size > 6; poor + rural + no school within 2 km) yielded effect sizes similar to those of the primary analysis (AOR range: 1.68–2.76) (Supplementary Table S8A).

*Entamoeba species adjustment:* assuming that 70–80% of microscopy-positive Entamoeba cases are non-pathogenic *E. dispar* (based on regional molecular epidemiology studies) reduced the overall IPI prevalence estimate by 33–40% but did not substantially alter the HDS–IPI association (Supplementary Table S8B).

*Deworming history:* the exclusion of children who received deworming medication in the past 6 months (*n =* 412) did not change the direction or magnitude of the HDS association (AOR = 2.94; 95% CI: 2.11–4.09; *p <* 0.001) (Supplementary Table S8G).

*Interviewer type:* restricting the analysis to children interviewed by external researchers (to minimize social desirability bias) strengthened the protective association of hand washing (AOR = 0.52; 95% CI: 0.38–0.71; *p <* 0.001) (Supplementary Table S8F).

*Missing data simulations:* worst-case scenario simulations (assuming 5–10% false negatives or non-response with higher infection rates) did not substantially change the HDS–IPI association (AOR range: 2.87–2.95) (Supplementary Table S8E).

*Design effect analysis:* the overall design effect (DEFF) for clustering was 1.12, indicating minimal variance inflation due to clustering. The effective sample size was 1,071 compared to the nominal 1,200 (Supplementary Table S18).

Restricting the interviewer type to external researchers strengthened the protective association of handwashing (AOR = 0.52; 95% CI: 0.38–0.71) (Supplementary Table S8F).

### 3.7 Exploratory burden of disease and cost-effectiveness modeling

Exploratory DALY and cost-effectiveness analyses, based on hypothetical modeling assumptions (50% DALY reduction, $0.50 per child), are presented in Supplementary File S9. These estimates are model-dependent, speculative, and should be interpreted with caution. Readers are referred to Supplementary File S9 for detailed calculations and sensitivity analyses.

## 4 Discussion

This study introduces a simple, scalable screening tool—the Household Deprivation Score (HDS)—for identifying schoolchildren at higher risk of intestinal parasitic infections in conflict-affected settings. The HDS, constructed from three simple questions about urban poverty, general poverty, and rural poverty, demonstrated a strong graded statistical association with IPI prevalence (p for trend < 0.001), with children in the highest deprivation category (HDS = 3) having nearly threefold higher odds of infection compared to those with no deprivation (AOR = 2.89).

Beyond the HDS, we identified several modifiable correlates statistically associated with IPI positivity: animals in the home (AOR = 1.89), irregular nail trimming (AOR = 1.82), anemia (AOR = 2.35), and infrequent handwashing (protective AOR = 0.63). These findings provide clear, actionable targets for low-cost interventions at the household and school levels.

### 4.1 Real-world impact: how can the HDS be used in practice?

The HDS is designed for frontline humanitarian settings where laboratory capacity is limited or non-existent. The tool requires no equipment, no blood draws, and no technical expertise—only the ability to ask three simple questions. We foresee the following practical applications:

- *For NGOs (e.g., Save the Children, Médecins Sans Frontières, International Rescue Committee):* the HDS can be integrated into rapid needs assessments to identify communities or schools where deworming campaigns should be prioritized when mass distribution is not feasible due to resource constraints.
- *For WHO programs and Ministries of Health:* the HDS can supplement existing WASH and neglected tropical disease (NTD) programs by providing a simple, standardized method for targeting school-based deworming, hygiene kit distribution, and nutrition support to the most vulnerable children.
- *For community health workers in the field:* a one-page scoring sheet (provided in Supplementary File S10) allows field workers to calculate an HDS score for each child in under 2 min and then follow a simple action checklist: deworming medication, iron supplementation, hygiene kit, and family education.

The tool is not intended to replace diagnostic testing for symptomatic children but rather to help allocate scarce resources equitably when universal coverage is impossible.

### 4.2 The household deprivation score (HDS) in context – a descriptive screening tool

The HDS combines three simple, easy-to-measure indicators. The graded association (p for trend < 0.001) is consistent with a syndemic perspective [13, 18], but the HDS is a descriptive, correlational measure. It provides a practical, low-cost tool for identifying high-risk populations without implying causality. Sensitivity analyses using non-overlapping definitions (Supplementary Table S8A) confirmed the robustness of this statistical association.

The Household Deprivation Score (HDS) shares conceptual similarities with existing vulnerability indices used in humanitarian research, such as the Multidimensional Poverty Index (MPI) [19] and the Deprivation Index used in conflict-affected settings [20]. However, unlike the MPI—which requires data collection on multiple domains (health, education, living standards) and typically takes 15–20 min to administer—the HDS was deliberately simplified to three binary questions that can be asked and scored in under 2 min by community health workers with no technical training. The HDS is, therefore, not intended to replace existing indices but to provide an ultra-low-cost, rapid triage tool for prioritizing interventions (deworming, nutrition support, hygiene kits) when universal coverage is impossible. External validation of the HDS in other humanitarian settings (Syria, Sudan, Afghanistan, Somalia) is required before widespread adoption.

### 4.3 Comparison with previous studies and the apparent protective association of hand washing before eating

Our findings align with previous research on individual correlates of IPIs [21–26]. The strong association between anemia and IPIs (AOR = 2.35) is consistent with the well-established bidirectional relationship [27–30].

The apparent protective association of handwashing before eating was examined by stratifying by interviewer type. The analysis strongly indicates social desirability bias (Supplementary Table S11). This finding has important methodological implications: external researchers may yield more accurate self-reported data than local health workers.

### 4.4 Conflict exposure: an unmeasured but conceptually relevant dimension

This study did not directly measure conflict exposure variables such as displacement history, exposure to violence, humanitarian aid receipt, or local conflict intensity. Therefore, we cannot determine whether the observed HDS–IPI association is driven by conflict-related mechanisms or pre-existing structural poverty. The term “conflict-affected setting” in the title describes the study context, not a measured exposure. Future research should incorporate direct measures of conflict exposure.

### 4.5 Strengths and limitations

*Strengths:*

- A large sample size (*N =* 1,200) with a multi-district design covering all nine districts of Al-Dhalea Governorate.
- Two diagnostic techniques increase diagnostic sensitivity.
- The first empirical validation of a simple cumulative household deprivation score (HDS) as a screening tool for IPI risk in a conflict-affected setting.
- Multilevel mixed-effects modeling accounts for clustering.
- Comprehensive sensitivity analyses (Supplementary File S8) confirm the robustness of the findings.
- Public data availability (OSF repository) enhances transparency.

*Limitations:*

1. Cross-sectional design precludes causal inference; all associations are correlational. The HDS is presented as a descriptive screening tool for risk stratification, not as a causal measure.
2. Microscopy cannot differentiate between *E. histolytica* and *E. dispar*. Sensitivity analyses suggest that true pathogenic amebiasis may be 33–40% lower (Supplementary Table S8B).
3. The HDS components are overlapping; non-overlapping sensitivity analyses are provided in Supplementary Table S8A.
4. The HDS does not measure conflict exposure, displacement history, or humanitarian aid receipt.
5. Recall and social desirability bias may affect self-reported behaviors; we adjusted for interviewer type to partially address this.
6. Generalizability is limited to similar conflict-affected settings; out-of-school children were not included.
7. DALY estimates are exploratory and model-dependent (Supplementary File S9). Readers should prioritize the cost-effectiveness ratio over the absolute DALY Figure.
8. The HDS is a novel tool developed for this study context. External validation in other conflict-affected settings (e.g., Syria, Sudan, Afghanistan, and Somalia) is required.
9. The HDS demonstrated only modest screening performance (AUC = 0.63, sensitivity = 67.2%, specificity = 61.4%), which limits its use as a stand-alone diagnostic tool. In resource-constrained settings, the 33% false negative rate means that one in three infected children would be missed using the HDS ≥ 2 cut-point. Therefore, the HDS is best used as a risk stratification tool to complement, not replace, clinical judgment and targeted testing.
10. Microscopy could not distinguish pathogenic *E. histolytica* from non-pathogenic *E. dispar*. Species-stratified analyses excluding Entamoeba cases showed substantial attenuation of the HDS-pathogen association (26.1% attenuation for HDS = 3; loss of statistical significance for HDS = 1, *p* = 0.108). Moreover, Entamoeba-only cases constituted 29.4% of all positive participants, raising the possibility that a substantial portion of the observed association is driven by non-pathogenic carriage. Future studies using PCR confirmation are urgently needed to validate the HDS against confirmed pathogenic infections.

### 4.6 Unanswered questions and future research

This study identifies several priority areas for future investigation:

1. Molecular diagnostics (PCR) are urgently needed to accurately distinguish *Entamoeba histolytica* from the non-pathogenic *E. dispar* and to determine the true prevalence of pathogenic amebiasis in this population.
2. Longitudinal studies incorporating direct measurements of conflict exposure, displacement history, humanitarian aid receipt, and household economic shocks are needed to understand the mechanisms linking deprivation to infection risk.
3. Out-of-school children, who were excluded from this study, may have an even higher IPI prevalence and should be prioritized in future surveys.
4. Monitoring for emerging anthelmintic resistance in Yemen is crucial as mass drug administration programs scale up.
5. Seasonal variation in IPI transmission should be characterized to optimize the timing of mass drug administration.
6. Validation of the HDS screening tool in other humanitarian settings (Syria, Sudan, Afghanistan, and Somalia) is required before widespread adoption.

### 4.7 Generalizability to other fragile settings

While this study was conducted in Yemen, its implications extend beyond this single context. The Household Deprivation Score (HDS) is constructed from simple, easy-to-measure indicators (poverty status and urban/rural residence) that are available in most low- and middle-income country (LMIC) settings. The graded association between cumulative deprivation and IPI prevalence observed here is likely to be present in other fragile and conflict-affected states, including Syria, Sudan, Afghanistan, and Somalia. The policy implications are therefore transferable: (1) school-based deworming is highly cost-effective under modeled assumptions; (2) household-level interventions (animal confinement, nail hygiene, and hand washing promotion) can be integrated into existing WASH programs; and (3) targeting interventions based on simple deprivation indicators can efficiently reach the most vulnerable populations.

## 5 Conclusion

The Household Deprivation Score (HDS) demonstrates a graded statistical association with intestinal parasitic infections among schoolchildren in a conflict-affected setting (AUC = 0.63, modest discriminative ability). Children with HDS ≥ 2 had approximately two- to three-fold higher odds of infection compared to those with no deprivation. However, the modest screening performance (sensitivity: 67.2%, specificity: 61.4%) indicates that the HDS is not suitable as a stand-alone diagnostic tool. Rather, it may serve as a pragmatic, low-cost risk stratification tool to help prioritize deworming, nutrition support, and hygiene education in humanitarian settings where universal coverage is impossible.

Critically, species-stratified analyses excluding Entamoeba cases (29.4% of positive participants, potentially non-pathogenic *E. dispar*) showed substantial attenuation of the HDS-pathogen association (26.1% attenuation for HDS = 3; loss of statistical significance for HDS = 1, *p* = 0.108). This suggests that part of the observed association is driven by non-pathogenic carriage and underscores the urgent need for PCR-based molecular confirmation to distinguish pathogenic *E. histolytica* from non-pathogenic *E. dispar* in future studies.

## Data Availability

The datasets presented in this study can be found in online repositories. The names of the repository/repositories and accession number(s) can be found in the article/Supplementary material.
