# Supplementary material for: Household deprivation score demonstrates graded association with intestinal parasitic infections among schoolchildren in a conflict-affected setting: a cross-sectional study
Source: Front Public Health. 2026 Jul 8;14:1868011. doi: 10.3389/fpubh.2026.1868011 (PMC13388386; doi:10.3389/fpubh.2026.1868011)
Supplement: Supplementary file 1 [file Supplementary_file_1.docx]

# File S1: STROBE Checklist and Conceptual framework

# Part 1: STROBE Statement -- Checklist of items that should be included in cross-sectional studies

**Study title:** Household Deprivation Score is a Scalable Screening Tool for Intestinal Parasitic Infections Among Schoolchildren in a Conflict-Affected Setting

| **Section/Item** | **Item No.** | **Recommendation** | **Reported on** |
| --- | --- | --- | --- |
| Title and abstract | 1a | Indicate the study's design with a commonly used term in the title | Title page |
|  | 1b | Provide in the abstract an informative summary of what was done and found | Abstract |
| **Introduction** |  |  |  |
| Background/rationale | 2 | Explain the scientific background and rationale for the investigation being reported | Introduction (paras 1-3) |
| Objectives | 3 | State specific objectives, including any prespecified hypotheses | Introduction (paras 4-5) |
| **Methods** |  |  |  |
| Study design | 4 | Present key elements of study design early | Methods, Section 2.1 |
| Setting | 5 | Describe the setting, locations, and relevant dates | Methods, Section 2.1 |
| Participants | 6a | Give the eligibility criteria, and the sources and methods of selection | Methods, Section 2.1 |
| Variables | 7 | Clearly define all outcomes, exposures, predictors, potential confounders, and effect modifiers | Methods, Sections 2.4-2.7; Table 1 |
| Data sources/measurement | 8 | For each variable, give the data sources and details of methods of assessment | Methods, Sections 2.3-2.7 |
| Bias | 9 | Describe any efforts to address potential sources of bias | Methods, Sections 2.5, 2.8; File S8 |
| Study size | 10 | Explain how the study size was arrived at | Methods, Section 2.2 |
| Quantitative variables | 11 | Explain how quantitative variables were handled | Methods, Section 2.8 |
| Statistical methods | 12a | Describe all statistical methods, including those used to control for confounding | Methods, Section 2.8 |
|  | 12b | Describe any methods used to examine subgroups and interactions | Methods, Section 2.8 |
|  | 12c | Explain how missing data were addressed | No missing data |
|  | 12d | Describe analytical methods accounting for sampling strategy | Methods, Section 2.2 (multi-stage cluster) |
|  | 12e | Describe any sensitivity analyses | Methods, Section 2.8; File S8 |
| **Results** |  |  |  |
| Participants | 13a | Report numbers of individuals at each stage of study | Results, Section 3.1 |
|  | 13b | Give reasons for non-participation | 100% response rate |
| Descriptive data | 14a | Give characteristics of study participants | Results, Table 2; File S3, Table S1 |
|  | 14b | Indicate number of participants with missing data | No missing data |
| Outcome data | 15 | Report numbers of outcome events or summary measures | Results, Section 3.2; Table 3 |
| Main results | 16a | Give unadjusted estimates and, if applicable, confounder-adjusted estimates | Results, Table 4 |
|  | 16b | Report category boundaries when continuous variables were categorized | Results, Table 2 |
|  | 16c | Consider translating estimates of relative risk into absolute risk | Results, Section 3.4; Figure 4 |
| Other analyses | 17 | Report other analyses done (subgroups, interactions, sensitivity) | Results, Section 3.5; File S8 |
| **Discussion** |  |  |  |
| Key results | 18 | Summarise key results with reference to study objectives | Discussion, first paragraph |
| Limitations | 19 | Discuss limitations of the study | Discussion, Section 4.4 |
| Interpretation | 20 | Give a cautious overall interpretation of results | Discussion, Sections 4.5-4.6 |
| Generalisability | 21 | Discuss the generalisability (external validity) of the study results | Discussion, Section 4.4 |
| **Other information** |  |  |  |
| Funding | 22 | Give the source of funding and the role of the funders | Declarations section |

# Part 2: Conceptual Framework Figure (Hypothesis‑Generating Only)

Figure 1: Conceptual framework illustrating the hypothesis‑generating approach examined in this study.

## IMPORTANT NOTES

- This figure represents a theoretical framework for hypothesis generation ONLY, not a causal model.
- All dashed lines represent theoretical associations examined as statistical correlations, NOT causal pathways.
- The cross-sectional design of this study COMPLETELY PRECLUDES any causal inference.
- The Household Deprivation Score (HDS) is a direct, descriptive measure of household-level socioeconomic and spatial deprivation. It is NOT a causal measure.
- All reported associations in this study are statistical correlations only. No causal claims are made or implied.
- Future research employing longitudinal designs, controlled interventions, or natural experiments is needed to test potential causal mechanisms.
- This framework is presented solely to guide hypothesis generation and to transparently illustrate the theoretical relationships that motivated the statistical analyses.

## ADDITIONAL CLARIFICATION FOR REVIEWERS AND READERS

The term "conflict-affected setting" in the title describes the study context, not a measured exposure. This study did not directly measure conflict exposure, displacement history, humanitarian aid receipt, or economic shocks at the household level. Readers should interpret all findings as correlational and context-specific to Al-Dhalea Governorate, Yemen, during the study period (April-October 2025).
