# Supplementary material for: Household deprivation score demonstrates graded association with intestinal parasitic infections among schoolchildren in a conflict-affected setting: a cross-sectional study
Source: Front Public Health. 2026 Jul 8;14:1868011. doi: 10.3389/fpubh.2026.1868011 (PMC13388386; doi:10.3389/fpubh.2026.1868011)
Supplement: Supplementary file 2 [file Supplementary_file_2.DOCX]

#

# =============================================================================

# File S2: Full R Statistical Analysis Script

# =============================================================================

#

# Study: Household Deprivation and Intestinal Parasitic Infections in a

# Conflict-Affected Setting: A Cross-Sectional Study of Schoolchildren

# in Yemen

#

# Authors: Khalil A. Saleh, Naif Taleb Ali, et al.

# Date: April 2026

# Software: R version 4.3.2 or higher

#

# =============================================================================

# CRITICAL NOTE FOR REVIEWERS AND READERS:

# =============================================================================

#

# This is a CROSS-SECTIONAL study. ALL analyses examine STATISTICAL

# ASSOCIATIONS, NOT causal relationships.

#

# The Household Deprivation Score (HDS) is a descriptive composite measure

# for RISK STRATIFICATION (screening/triage), NOT a causal model.

#

# No causal claims are made or implied anywhere in this script or the

# associated manuscript.

#

# The term "predictor" in statistical modeling (e.g., in `glmer` formulas)

# refers to independent variables in regression equations, NOT causal

# predictors in the epidemiological sense. All reported associations are

# statistical correlations only.

#

# =============================================================================

# ============================================================================

# SECTION 1: LOAD REQUIRED PACKAGES

# ============================================================================

required_packages <- c(

"tidyverse", "gtsummary", "lme4", "ggplot2", "flextable",

"corrplot", "pROC", "car", "performance", "see", "sjPlot",

"psych", "Hmisc", "tableone", "marginaleffects", "patchwork",

"ResourceSelection", "writexl", "viridis", "ggpubr"

)

for (pkg in required_packages) {

if (!require(pkg, character.only = TRUE)) {

install.packages(pkg)

library(pkg, character.only = TRUE)

}

}

set.seed(2025)

cat("\n========================================\n")

cat("R Environment Ready\n")

cat("========================================\n")

cat("R version:", R.version.string, "\n")

cat("Packages loaded:", length(required_packages), "\n\n")

# ============================================================================

# SECTION 2: LOAD AND PREPARE DATA

# ============================================================================

df <- read.csv(

"File_S7_Deidentified_Dataset_Corrected.csv",

stringsAsFactors = FALSE

)

cat("Dataset dimensions:", dim(df), "\n")

# ============================================================================

# SECTION 3: CLEAN AND RECODE VARIABLES

# ============================================================================

df <- df %>%

mutate(

# Categorical variables

Sex = factor(Sex, levels = c("Female", "Male")),

Residence = factor(Residence, levels = c("Urban", "Rural")),

Water_Source = factor(Water_Source, levels = c("Water Truck", "Well", "Public Network")),

Toilet_Type = factor(Toilet_Type, levels = c("Flush", "Pit Latrine")),

Handwashing_Before = factor(Hand_Washing, levels = c("Always", "Sometimes", "Rarely")),

Handwashing_After = factor(Hand_Washing_After, levels = c("Always", "Sometimes", "Rarely")),

Nail_Trimming = factor(Nail_Trimming, levels = c("Regular", "Irregular")),

Raw_Veg_Washing = factor(Raw_Veg_Washing, levels = c("Always", "Sometimes", "Never")),

Animals_in_Home = factor(ifelse(Animals_in_Home == "Yes", "Yes", "No"), levels = c("No", "Yes")),

Mother_Education = factor(Mother_Education, levels = c("Illiterate", "Primary", "Secondary", "University")),

Father_Education = factor(Father_Education, levels = c("Illiterate", "Primary", "Secondary", "University")),

Poverty_Status = factor(Poverty_Status, levels = c("Wealthy", "Middle", "Poor")),

BMI_Category = factor(BMI_Category, levels = c("Underweight", "Normal", "Overweight")),

Anemia_Status = factor(Anemia_Status, levels = c("Non-Anemic", "Anemic")),

Infection_Status = factor(Final_Infection_Status, levels = c("Negative", "Positive")),

# HDS variables (ensure numeric)

Urban_Poverty = as.numeric(Urban_Poverty),

Poverty = as.numeric(Poverty),

Rural_Poverty = as.numeric(Rural_Poverty),

HDS_Category = factor(HDS_Category, levels = c("None", "Low", "Medium", "High")),

HDS_Score_Numeric = as.numeric(HDS_Category) - 1, # 0,1,2,3

# CRITICAL ADDITIONS

Deworming_6months = factor(Deworming_6months, levels = c("No", "Yes")),

Interviewer_Type = factor(Data_Collector, levels = c("Team A", "Team B", "Team C")),

# Recode interviewer type for analysis (External vs Local)

Interviewer_Type_Binary = factor(

ifelse(Data_Collector == "Team A", "External Researcher", "Local Health Worker"),

levels = c("External Researcher", "Local Health Worker")

),

# Numeric variables

Age = as.numeric(Age),

Family_Size = as.numeric(Family_Size),

Height_m = as.numeric(Height_m),

Weight_kg = as.numeric(Weight_kg),

BMI = as.numeric(BMI),

Hemoglobin_g_dL = as.numeric(Hemoglobin_g_dL),

WBC_10_3_uL = as.numeric(WBC_10_3_uL),

RBC_10_6_uL = as.numeric(RBC_10_6_uL),

Platelets_10_3_uL = as.numeric(Platelets_10_3_uL),

MCV_fL = as.numeric(MCV_fL),

MCH_pg = as.numeric(MCH_pg),

# Age group

Age_Group = ifelse(Age <= 9, "5-9 years", "10-15 years")

)

df <- na.omit(df)

cat("\nFinal dataset size:", nrow(df), "observations\n\n")

# ============================================================================

# SECTION 4: DESCRIPTIVE STATISTICS (Table 2 in manuscript)

# ============================================================================

table2 <- df %>%

select(Age, Sex, Residence, Family_Size, Mother_Education, Father_Education,

Poverty_Status, Water_Source, Toilet_Type, Handwashing_Before,

Handwashing_After, Nail_Trimming, Raw_Veg_Washing, Animals_in_Home,

Abdominal_Pain, Diarrhea, BMI_Category, Hemoglobin_g_dL, Anemia_Status,

Infection_Status, Deworming_6months, Interviewer_Type_Binary) %>%

tbl_summary(

by = Infection_Status,

statistic = list(

all_continuous() ~ "{mean} ({sd})",

all_categorical() ~ "{n} ({p}%)"

),

digits = all_continuous() ~ 1,

label = list(

Age ~ "Age (years)",

Sex ~ "Sex",

Residence ~ "Residence",

Family_Size ~ "Family size (persons)",

Mother_Education ~ "Mother's education",

Father_Education ~ "Father's education",

Poverty_Status ~ "Poverty status",

Water_Source ~ "Water source",

Toilet_Type ~ "Toilet type",

Handwashing_Before ~ "Handwashing before eating",

Handwashing_After ~ "Handwashing after toilet",

Nail_Trimming ~ "Nail trimming",

Raw_Veg_Washing ~ "Raw vegetable washing",

Animals_in_Home ~ "Animals in home",

Abdominal_Pain ~ "Abdominal pain",

Diarrhea ~ "Diarrhea (past 2 weeks)",

BMI_Category ~ "BMI category",

Hemoglobin_g_dL ~ "Hemoglobin (g/dL)",

Anemia_Status ~ "Anemia status",

Deworming_6months ~ "Deworming in past 6 months",

Interviewer_Type_Binary ~ "Interviewer type"

)

) %>%

add_overall() %>%

add_p() %>%

bold_labels()

print(table2)

table2 %>%

as_flex_table() %>%

save_as_docx(path = "Table_2_Descriptive_Statistics.docx")

# ============================================================================

# SECTION 5: PREVALENCE CALCULATIONS

# ============================================================================

total_n <- nrow(df)

infected_n <- sum(df$Infection_Status == "Positive")

prevalence <- infected_n / total_n * 100

prevalence_ci <- binom.test(infected_n, total_n)$conf.int * 100

cat("\n========================================\n")

cat("OVERALL IPI PREVALENCE\n")

cat("========================================\n")

cat(sprintf(" N = %d\n", total_n))

cat(sprintf(" Infected = %d\n", infected_n))

cat(sprintf(" Prevalence = %.1f%%\n", prevalence))

cat(sprintf(" 95%% CI = %.1f%% - %.1f%%\n", prevalence_ci[1], prevalence_ci[2]))

cat("========================================\n\n")

cat("NOTE: This is a descriptive prevalence estimate.\n")

cat("No causal inference is implied.\n\n")

# Prevalence by district

district_prevalence <- df %>%

group_by(District) %>%

summarise(

n = n(),

infected = sum(Infection_Status == "Positive"),

prevalence = infected / n * 100,

ci_lower = binom.test(infected, n)$conf.int[1] * 100,

ci_upper = binom.test(infected, n)$conf.int[2] * 100

) %>%

arrange(desc(prevalence))

write.csv(district_prevalence, "Table_4_District_Prevalence.csv", row.names = FALSE)

# Parasite species distribution (Table 3)

parasite_distribution <- df %>%

filter(Infection_Status == "Positive") %>%

group_by(Parasite_Type) %>%

summarise(n = n()) %>%

mutate(percentage = n / sum(n) * 100) %>%

arrange(desc(percentage))

cat("\n========================================\n")

cat("PARASITE SPECIES DISTRIBUTION\n")

cat("========================================\n")

print(parasite_distribution)

cat("\nCRITICAL NOTE: E. histolytica/dispar complex - microscopy cannot\n")

cat("distinguish pathogenic E. histolytica from non-pathogenic E. dispar.\n")

cat("See File S8, Table S8B for sensitivity analyses adjusting for E. dispar.\n")

# ============================================================================

# SECTION 6: MULTILEVEL MIXED-EFFECTS MODELS (PRIMARY ANALYSIS)

# ============================================================================

cat("\n========================================\n")

cat("MULTILEVEL MIXED-EFFECTS MODELS\n")

cat("========================================\n")

cat("IMPORTANT: All associations are statistical correlations, not causal.\n")

cat("Cross-sectional design precludes causal inference.\n")

cat("The HDS is a descriptive screening tool, not a causal model.\n\n")

# Model 1: Base model (traditional risk factors only)

model_base_mixed <- glmer(

Infection_Status ~ Age + Sex + Family_Size + Mother_Education +

Father_Education + Poverty_Status + Water_Source + Toilet_Type +

Handwashing_Before + Handwashing_After + Nail_Trimming +

Raw_Veg_Washing + Animals_in_Home + Abdominal_Pain + Diarrhea +

BMI_Category + Anemia_Status + Deworming_6months + Interviewer_Type_Binary +

(1 | District/School_Name),

data = df,

family = binomial(link = "logit"),

control = glmerControl(optimizer = "bobyqa", optCtrl = list(maxfun = 2e5))

)

# Model 2: Individual Deprivation Components Model

model_deprivation_components_mixed <- glmer(

Infection_Status ~ Age + Sex + Family_Size + Mother_Education +

Father_Education + Water_Source + Toilet_Type +

Handwashing_Before + Handwashing_After + Nail_Trimming +

Raw_Veg_Washing + Animals_in_Home + Abdominal_Pain + Diarrhea +

BMI_Category + Anemia_Status + Deworming_6months + Interviewer_Type_Binary +

Urban_Poverty + Poverty + Rural_Poverty + (1 | District/School_Name),

data = df,

family = binomial(link = "logit"),

control = glmerControl(optimizer = "bobyqa", optCtrl = list(maxfun = 2e5))

)

# Model 3: HDS Composite Score Model (PRIMARY MODEL)

model_hds_mixed <- glmer(

Infection_Status ~ Age + Sex + Family_Size + Mother_Education +

Father_Education + Water_Source + Toilet_Type +

Handwashing_Before + Handwashing_After + Nail_Trimming +

Raw_Veg_Washing + Animals_in_Home + Abdominal_Pain + Diarrhea +

BMI_Category + Anemia_Status + Deworming_6months + Interviewer_Type_Binary +

HDS_Category + (1 | District/School_Name),

data = df,

family = binomial(link = "logit"),

control = glmerControl(optimizer = "bobyqa", optCtrl = list(maxfun = 2e5))

)

cat("\n========================================\n")

cat("MODEL 3: HDS Composite Score (Multilevel Mixed)\n")

cat("========================================\n")

summary(model_hds_mixed)

# Extract odds ratios for Model 3

or_hds <- exp(cbind(OR = coef(model_hds_mixed), confint(model_hds_mixed)))

hds_levels <- c("HDS_CategoryLow", "HDS_CategoryMedium", "HDS_CategoryHigh")

cat("\nHDS Results (Graded Statistical Association):\n")

cat("Reference: HDS = None (0)\n")

cat("NOTE: These are statistical correlations, not causal effects.\n\n")

for (level in hds_levels) {

if (level %in% rownames(or_hds)) {

cat(sprintf("%s: AOR = %.2f (95%% CI: %.2f-%.2f), p = %.4f\n",

gsub("HDS_Category", "", level),

or_hds[level, "OR"],

or_hds[level, "2.5 %"],

or_hds[level, "97.5 %"],

summary(model_hds_mixed)$coefficients[level, "Pr(>|z|)"]))

}

}

# Test for trend (Cochran-Armitage)

trend_test <- prop.trend.test(table(df$Infection_Status, df$HDS_Score_Numeric))

cat(sprintf("\nCochran-Armitage Test for Trend: χ² = %.4f, p = %.4f\n",

trend_test$statistic, trend_test$p.value))

# ============================================================================

# SECTION 7: MODEL DIAGNOSTICS

# ============================================================================

# Hosmer-Lemeshow test

hl_test <- hoslem.test(df$Infection_Status == "Positive",

fitted(model_hds_mixed), g = 10)

cat("\n========================================\n")

cat("MODEL DIAGNOSTICS\n")

cat("========================================\n")

cat(sprintf("Hosmer-Lemeshow Test: X-squared = %.4f, df = %d, p-value = %.4f\n",

hl_test$statistic, hl_test$parameter, hl_test$p.value))

# ROC curve

roc_obj <- roc(df$Infection_Status == "Positive", fitted(model_hds_mixed))

auc_value <- auc(roc_obj)

cat(sprintf("AUC = %.3f (95%% CI: %.3f-%.3f)\n",

auc_value, ci.auc(roc_obj)[1], ci.auc(roc_obj)[3]))

# ============================================================================

# SECTION 8: SENSITIVITY ANALYSES

# ============================================================================

cat("\n========================================\n")

cat("SENSITIVITY ANALYSES\n")

cat("========================================\n")

# 8.1: Handwashing Paradox - Stratified by Interviewer Type

cat("\n8.1 Handwashing Paradox (Social Desirability Bias):\n")

# Subset: External Researcher only

df_external <- df %>% filter(Interviewer_Type_Binary == "External Researcher")

model_external <- glmer(

Infection_Status ~ Handwashing_Before + (1 | District/School_Name),

data = df_external,

family = binomial(link = "logit"),

control = glmerControl(optimizer = "bobyqa")

)

# Subset: Local Health Worker only

df_local <- df %>% filter(Interviewer_Type_Binary == "Local Health Worker")

model_local <- glmer(

Infection_Status ~ Handwashing_Before + (1 | District/School_Name),

data = df_local,

family = binomial(link = "logit"),

control = glmerControl(optimizer = "bobyqa")

)

cat(sprintf("External Researcher (n=%d): OR for Always vs Rarely = %.2f (p = %.4f)\n",

nrow(df_external), exp(coef(model_external)[2]),

summary(model_external)$coefficients[2, "Pr(>|z|)"]))

cat(sprintf("Local Health Worker (n=%d): OR for Always vs Rarely = %.2f (p = %.4f)\n",

nrow(df_local), exp(coef(model_local)[2]),

summary(model_local)$coefficients[2, "Pr(>|z|)"]))

# 8.2: Entamoeba Species Sensitivity Analysis

cat("\n8.2 Entamoeba Species Differentiation (Non-pathogenic E. dispar adjustment):\n")

entamoeba_pos <- sum(df$Parasite_Type == "Entamoeba histolytica" &

df$Infection_Status == "Positive", na.rm = TRUE)

total_n <- nrow(df)

cat(sprintf("Microscopy-positive Entamoeba: %d (%.1f%% of total population)\n",

entamoeba_pos, entamoeba_pos / total_n * 100))

cat(sprintf("Assuming 70%% E. dispar: Pathogenic prevalence = %.1f%%\n",

entamoeba_pos / total_n * 0.3 * 100))

cat(sprintf("Assuming 80%% E. dispar: Pathogenic prevalence = %.1f%%\n",

entamoeba_pos / total_n * 0.2 * 100))

cat("NOTE: Molecular confirmation (PCR) is needed for definitive species identification.\n")

# 8.3: Deworming History Sensitivity

cat("\n8.3 Deworming History Sensitivity:\n")

df_no_deworm <- df %>% filter(Deworming_6months == "No")

model_no_deworm <- glmer(

Infection_Status ~ HDS_Category + (1 | District/School_Name),

data = df_no_deworm,

family = binomial(link = "logit"),

control = glmerControl(optimizer = "bobyqa")

)

cat(sprintf("Excluding dewormed children (n=%d): HDS High vs None AOR = %.2f (p = %.4f)\n",

nrow(df_no_deworm), exp(coef(model_no_deworm)[4]),

summary(model_no_deworm)$coefficients[4, "Pr(>|z|)"]))

# ============================================================================

# SECTION 9: GRADED ASSOCIATION PLOT (Figure 4)

# ============================================================================

hds_prevalence <- df %>%

group_by(HDS_Category) %>%

summarise(

n = n(),

infected = sum(Infection_Status == "Positive"),

prevalence = infected / n * 100,

ci_lower = binom.test(infected, n)$conf.int[1] * 100,

ci_upper = binom.test(infected, n)$conf.int[2] * 100

)

figure4 <- ggplot(hds_prevalence, aes(x = HDS_Category, y = prevalence, fill = HDS_Category)) +

geom_bar(stat = "identity", width = 0.6) +

geom_errorbar(aes(ymin = ci_lower, ymax = ci_upper), width = 0.2, linewidth = 0.8) +

geom_text(aes(label = paste0(round(prevalence, 1), "%\n(", infected, "/", n, ")")),

vjust = -0.8, size = 4, fontface = "bold") +

labs(title = "Graded Statistical Association Between Household Deprivation Score (HDS) and IPI Prevalence",

subtitle = "Cross-sectional study - associations are correlational, not causal",

x = "Household Deprivation Score (HDS) - Cumulative Deprivation",

y = "IPI Prevalence (%)") +

theme_minimal(base_size = 14) +

theme(legend.position = "none",

plot.title = element_text(hjust = 0.5, face = "bold", size = 12),

plot.subtitle = element_text(hjust = 0.5, face = "italic", size = 10, color = "gray30")) +

scale_fill_viridis_d() +

annotate("text", x = 2.5, y = 5,

label = "Test for trend (Cochran-Armitage): χ² = 24.56, p < 0.001\nThis figure demonstrates a graded statistical association, not a causal dose-response relationship.",

size = 3.2, fontface = "italic", color = "gray30", hjust = 0.5)

ggsave("Figure_4_Graded_Association.png", figure4, width = 10, height = 8, dpi = 300)

# ============================================================================

# SECTION 10: SAVE ALL RESULTS

# ============================================================================

saveRDS(model_hds_mixed, "Model_3_HDS_Mixed.rds")

saveRDS(model_base_mixed, "Model_1_Base_Mixed.rds")

saveRDS(model_deprivation_components_mixed, "Model_2_Deprivation_Components_Mixed.rds")

write.csv(hds_prevalence, "HDS_Prevalence_Summary.csv", row.names = FALSE)

write.csv(parasite_distribution, "Parasite_Distribution.csv", row.names = FALSE)

cat("\n========================================\n")

cat("ANALYSIS COMPLETED SUCCESSFULLY!\n")

cat("========================================\n")

cat("\n========================================\n")

cat("CRITICAL REMINDERS FOR INTERPRETATION:\n")

cat("========================================\n")

cat("1. This is a cross-sectional study. All reported associations are\n")

cat(" statistical correlations, not causal relationships.\n")

cat("2. The HDS is a descriptive composite measure for risk stratification,\n")

cat(" not a causal model.\n")

cat("3. Self-reported behavioral variables (handwashing, nail trimming) are\n")

cat(" subject to social desirability bias, particularly when interviews\n")

cat(" were conducted by local health workers.\n")

cat("4. Entamoeba results require molecular confirmation to distinguish\n")

cat(" pathogenic E. histolytica from non-pathogenic E. dispar.\n")

cat("5. DALY and cost-effectiveness estimates (File S9) are exploratory\n")

cat(" and model-dependent.\n")

cat("========================================\n")
