# Supplementary material for: Household deprivation score demonstrates graded association with intestinal parasitic infections among schoolchildren in a conflict-affected setting: a cross-sectional study
Source: Front Public Health. 2026 Jul 8;14:1868011. doi: 10.3389/fpubh.2026.1868011 (PMC13388386; doi:10.3389/fpubh.2026.1868011)
Supplement: Supplementary file 3 [file Supplementary_file_3.DOCX]

**File S3: Supplementary Tables and Figures**

**Table S1: Detailed Baseline Characteristics by District (N = 1,200)**

| Characteristic | Al-Azariq (n=133) | Al-Dhalea (n=134) | Jahaf (n=132) | Al-Shaib (n=134) | Al-Husain (n=133) | Qa'tabah (n=134) | Juban (n=133) | Al-Hasha (n=134) | Damt (n=133) |
| --- | --- | --- | --- | --- | --- | --- | --- | --- | --- |
| Age, years (mean ± SD) | 10.3 ± 3.0 | 10.6 ± 3.2 | 10.4 ± 3.1 | 10.5 ± 3.0 | 10.7 ± 3.2 | 10.4 ± 3.1 | 10.6 ± 3.3 | 10.5 ± 3.0 | 10.4 ± 3.1 |
| Sex (% female) | 49.6 | 50.7 | 49.2 | 50.0 | 51.1 | 49.3 | 51.1 | 50.0 | 50.4 |
| Residence (% rural) | 42.1 | 44.8 | 53.0 | 46.3 | 48.9 | 44.0 | 42.9 | 47.0 | 45.9 |
| Family size (mean ± SD) | 6.3 ± 2.5 | 6.5 ± 2.7 | 6.6 ± 2.6 | 6.4 ± 2.5 | 6.5 ± 2.6 | 6.3 ± 2.5 | 6.2 ± 2.4 | 6.5 ± 2.7 | 6.4 ± 2.6 |
| **Mother's education (%)** |  |  |  |  |  |  |  |  |  |
| Illiterate | 39.8 | 41.0 | 43.9 | 41.0 | 42.1 | 41.0 | 40.6 | 41.8 | 41.4 |
| Primary | 26.3 | 26.1 | 25.8 | 26.1 | 26.3 | 26.1 | 26.3 | 26.1 | 25.6 |
| Secondary | 20.3 | 19.4 | 18.9 | 19.4 | 19.5 | 19.4 | 19.5 | 19.4 | 19.5 |
| University | 13.6 | 13.4 | 11.4 | 13.4 | 12.0 | 13.4 | 13.5 | 12.7 | 13.5 |
| **Father's education (%)** |  |  |  |  |  |  |  |  |  |
| Illiterate | 31.6 | 32.1 | 34.1 | 32.1 | 33.1 | 32.1 | 31.6 | 32.8 | 31.6 |
| Primary | 23.3 | 23.1 | 22.7 | 23.1 | 23.3 | 23.1 | 23.3 | 23.1 | 22.6 |
| Secondary | 22.6 | 22.4 | 21.2 | 22.4 | 21.8 | 22.4 | 22.6 | 22.4 | 22.6 |
| University | 22.6 | 22.4 | 22.0 | 22.4 | 21.8 | 22.4 | 22.6 | 21.6 | 23.3 |
| **Poverty status (%)** |  |  |  |  |  |  |  |  |  |
| Wealthy | 13.5 | 12.7 | 12.1 | 12.7 | 13.5 | 13.4 | 13.5 | 12.7 | 12.8 |
| Middle | 32.3 | 32.1 | 31.8 | 32.1 | 32.3 | 32.8 | 32.3 | 32.8 | 32.3 |
| Poor | 54.1 | 55.2 | 56.1 | 55.2 | 54.1 | 53.7 | 54.1 | 54.5 | 54.9 |
| **Water source (%)** |  |  |  |  |  |  |  |  |  |
| Water truck | 48.9 | 49.3 | 50.8 | 49.3 | 50.4 | 50.0 | 49.6 | 50.7 | 50.4 |
| Well | 28.6 | 27.6 | 28.0 | 27.6 | 27.8 | 27.6 | 27.8 | 27.6 | 27.8 |
| Public network | 22.6 | 23.1 | 21.2 | 23.1 | 21.8 | 22.4 | 22.6 | 21.6 | 21.8 |
| Toilet type (% pit latrine) | 50.4 | 51.5 | 53.0 | 51.5 | 52.6 | 51.5 | 50.4 | 52.2 | 51.9 |
| **Handwashing before eating (%)** |  |  |  |  |  |  |  |  |  |
| Always | 31.6 | 31.3 | 30.3 | 31.3 | 31.6 | 32.1 | 31.6 | 31.3 | 31.6 |
| Sometimes | 42.9 | 43.3 | 43.2 | 43.3 | 43.6 | 42.5 | 43.6 | 43.3 | 42.9 |
| Rarely | 25.6 | 25.4 | 26.5 | 25.4 | 24.8 | 25.4 | 24.8 | 25.4 | 25.6 |
| **Handwashing after toilet (%)** |  |  |  |  |  |  |  |  |  |
| Always | 29.3 | 29.9 | 28.8 | 29.9 | 30.1 | 29.9 | 29.3 | 29.9 | 29.3 |
| Sometimes | 40.6 | 41.0 | 40.2 | 41.0 | 41.4 | 40.3 | 41.4 | 40.3 | 40.6 |
| Rarely | 30.1 | 29.1 | 31.1 | 29.1 | 28.6 | 29.9 | 29.3 | 29.9 | 30.1 |
| Nail trimming (% irregular) | 48.9 | 50.0 | 51.5 | 50.0 | 51.1 | 50.0 | 48.9 | 50.0 | 49.6 |
| **Raw vegetable washing (%)** |  |  |  |  |  |  |  |  |  |
| Always | 44.4 | 44.0 | 43.9 | 44.0 | 45.1 | 44.8 | 44.4 | 44.8 | 44.4 |
| Sometimes | 35.3 | 35.1 | 35.6 | 35.1 | 35.3 | 34.3 | 35.3 | 35.1 | 35.3 |
| Never | 20.3 | 20.9 | 20.5 | 20.9 | 19.5 | 20.9 | 20.3 | 20.1 | 20.3 |
| Animals in home (% yes) | 48.1 | 49.3 | 52.3 | 49.3 | 51.1 | 49.3 | 48.1 | 50.0 | 49.6 |
| Abdominal pain (% yes) | 40.6 | 41.8 | 43.2 | 41.8 | 42.9 | 41.0 | 40.6 | 41.0 | 40.6 |
| Diarrhea (% yes) | 27.1 | 27.6 | 29.5 | 27.6 | 28.6 | 27.6 | 27.1 | 28.4 | 27.1 |
| **BMI category (%)** |  |  |  |  |  |  |  |  |  |
| Underweight | 60.9 | 61.9 | 63.6 | 61.9 | 62.4 | 61.9 | 60.9 | 61.9 | 61.7 |
| Normal | 34.6 | 33.6 | 33.3 | 33.6 | 33.8 | 34.3 | 34.6 | 34.3 | 34.6 |
| Overweight | 4.5 | 4.5 | 3.0 | 4.5 | 3.8 | 3.7 | 4.5 | 3.7 | 3.8 |
| Anemia (% anemic) | 32.3 | 33.6 | 35.6 | 33.6 | 34.6 | 32.8 | 32.3 | 33.6 | 32.3 |
| Hemoglobin (g/dL, mean ± SD) | 11.9 ± 1.6 | 11.8 ± 1.6 | 11.7 ± 1.7 | 11.8 ± 1.6 | 11.8 ± 1.6 | 11.9 ± 1.6 | 11.9 ± 1.6 | 11.8 ± 1.6 | 11.9 ± 1.5 |

**Table S2: Bivariate Analysis (Chi-Square Tests) – All Variables (N = 1,200)**

| **Variable** | **Category** | **Total N** | **Infected n** | **Infection Rate (%)** | **χ²** | **df** | **p-value** |
| --- | --- | --- | --- | --- | --- | --- | --- |
| Age group | 5–9 years | 498 | 215 | 43.2 | 5.62 | 1 | 0.018 |
|  | 10–15 years | 702 | 340 | 48.4 |  |  |  |
| Sex | Female | 602 | 272 | 45.2 | 0.56 | 1 | 0.454 |
|  | Male | 598 | 283 | 47.3 |  |  |  |
| Residence | Urban | 645 | 289 | 44.8 | 1.24 | 1 | 0.266 |
|  | Rural | 555 | 266 | 47.9 |  |  |  |
| Family size | Small (2–5) | 498 | 212 | 42.6 | 10.94 | 2 | 0.004 |
|  | Medium (6–8) | 456 | 220 | 48.2 |  |  |  |
|  | Large (≥9) | 246 | 123 | 50.0 |  |  |  |
| Mother's education | Illiterate | 498 | 250 | 50.2 | 11.62 | 3 | 0.021 |
|  | Primary | 312 | 143 | 45.8 |  |  |  |
|  | Secondary | 234 | 98 | 41.9 |  |  |  |
|  | University | 156 | 64 | 41.0 |  |  |  |
| Father's education | Illiterate | 389 | 198 | 50.9 | 10.54 | 3 | 0.032 |
|  | Primary | 278 | 128 | 46.0 |  |  |  |
|  | Secondary | 267 | 116 | 43.4 |  |  |  |
|  | University | 266 | 113 | 42.5 |  |  |  |
| Poverty status | Wealthy | 156 | 60 | 38.5 | 11.76 | 2 | 0.008 |
|  | Middle | 389 | 170 | 43.7 |  |  |  |
|  | Poor | 655 | 325 | 49.6 |  |  |  |
| Water source | Water truck | 600 | 260 | 43.3 | 10.92 | 2 | 0.012 |
|  | Well | 334 | 168 | 50.3 |  |  |  |
|  | Public network | 266 | 127 | 47.7 |  |  |  |
| Toilet type | Flush | 578 | 247 | 42.7 | 5.93 | 1 | 0.015 |
|  | Pit latrine | 622 | 308 | 49.5 |  |  |  |
| Handwashing before eating | Always | 378 | 139 | 36.8 | 27.74 | 2 | <0.001 |
|  | Sometimes | 518 | 255 | 49.2 |  |  |  |
|  | Rarely | 304 | 161 | 53.0 |  |  |  |
| Handwashing after toilet | Always | 356 | 145 | 40.7 | 12.06 | 2 | 0.002 |
|  | Sometimes | 489 | 235 | 48.1 |  |  |  |
|  | Rarely | 355 | 175 | 49.3 |  |  |  |
| Nail trimming | Regular | 600 | 245 | 40.8 | 16.53 | 1 | <0.001 |
|  | Irregular | 600 | 310 | 51.7 |  |  |  |
| Raw vegetable washing | Always | 534 | 232 | 43.4 | 7.60 | 2 | 0.022 |
|  | Sometimes | 422 | 205 | 48.6 |  |  |  |
|  | Never | 244 | 118 | 48.4 |  |  |  |
| Animals in home | No | 602 | 227 | 37.7 | 36.04 | 1 | <0.001 |
|  | Yes | 598 | 328 | 54.8 |  |  |  |
| Abdominal pain* | No | 702 | 294 | 41.9 | 15.06 | 1 | <0.001 |
|  | Yes | 498 | 261 | 52.4 |  |  |  |
| Diarrhea (past 2 weeks)* | No | 866 | 382 | 44.1 | 8.66 | 1 | 0.003 |
|  | Yes | 334 | 173 | 51.8 |  |  |  |
| BMI category | Underweight | 741 | 358 | 48.3 | 7.16 | 2 | 0.028 |
|  | Normal | 408 | 176 | 43.1 |  |  |  |
|  | Overweight | 51 | 21 | 41.2 |  |  |  |
| Anemia status | Non-anemic | 800 | 318 | 39.8 | 49.55 | 1 | <0.001 |
|  | Anemic | 400 | 237 | 59.2 |  |  |  |
| Deworming (past 6 months) | No | 788 | 399 | 50.6 | 42.18 | 1 | <0.001 |
|  | Yes | 412 | 156 | 37.9 |  |  |  |
| Interviewer type | External researcher | 456 | 188 | 41.2 | 7.56 | 1 | 0.006 |
|  | Local health worker | 744 | 367 | 49.3 |  |  |  |

*Important note: Variables marked with an asterisk (*) – abdominal pain and diarrhea – may be consequences of infection rather than independent risk factors. Their inclusion in multivariable models is exploratory and should be interpreted with caution. The cross-sectional design precludes determination of temporality.*

*For continuous variables (age, haemoglobin, WBC, RBC, platelets, MCV, MCH) presented in other tables (e.g., Table S3), p‑values for comparisons between infected and non‑infected groups were calculated using the Wilcoxon rank‑sum test (Mann‑Whitney U test) as normality assumptions were not satisfied. For categorical variables in this table, the chi‑square test was used.*

**Table S3: Correlation Matrix of Continuous Variables (N = 1,200)**

| **Variable** | **Age** | **Family Size** | **Height (m)** | **Weight (kg)** | **BMI** | **Hemoglobin** | **WBC** | **RBC** | **Platelets** | **MCV** | **MCH** |
| --- | --- | --- | --- | --- | --- | --- | --- | --- | --- | --- | --- |
| Age | 1.00 | 0.08 | 0.85 | 0.79 | 0.12 | 0.15 | -0.03 | 0.18 | -0.05 | 0.22 | 0.19 |
| Family Size | 0.08 | 1.00 | 0.05 | 0.07 | 0.04 | -0.09 | 0.06 | -0.04 | 0.03 | 0.02 | 0.01 |
| Height (m) | 0.85 | 0.05 | 1.00 | 0.88 | 0.11 | 0.12 | -0.02 | 0.16 | -0.04 | 0.24 | 0.21 |
| Weight (kg) | 0.79 | 0.07 | 0.88 | 1.00 | 0.56 | 0.10 | -0.01 | 0.14 | -0.03 | 0.20 | 0.18 |
| BMI | 0.12 | 0.04 | 0.11 | 0.56 | 1.00 | 0.02 | 0.01 | 0.03 | 0.01 | 0.02 | 0.01 |
| Hemoglobin | 0.15 | -0.09 | 0.12 | 0.10 | 0.02 | 1.00 | 0.08 | 0.45 | 0.12 | 0.35 | 0.38 |
| WBC | -0.03 | 0.06 | -0.02 | -0.01 | 0.01 | 0.08 | 1.00 | 0.22 | 0.31 | -0.02 | -0.01 |
| RBC | 0.18 | -0.04 | 0.16 | 1.14 | 0.03 | 0.45 | 0.22 | 1.00 | 0.18 | 0.28 | 0.25 |
| Platelets | -0.05 | 0.03 | -0.04 | -0.03 | 0.01 | 0.12 | 0.31 | 0.18 | 1.00 | -0.04 | -0.02 |
| MCV | 0.22 | 0.02 | 0.24 | 0.20 | 0.02 | 0.35 | -0.02 | 0.28 | -0.04 | 1.00 | 0.88 |
| MCH | 0.19 | 0.01 | 0.21 | 0.18 | 0.01 | 0.38 | -0.01 | 0.25 | -0.02 | 0.88 | 1.00 |

*Note: **p < 0.01; *p < 0.05. Pearson correlation coefficients reported.*

*Important methodological note: High correlations between age, height, and weight (r ≥ 0.79) are expected due to biological growth patterns. To avoid multicollinearity, these variables were not included simultaneously in the same regression model. Only age was retained as a covariate in the final multivariable models presented in the main manuscript (Table 4). Sensitivity analyses replacing age with height-for-age Z-scores yielded similar results (data not shown).*

*For comparisons of continuous variables between infected and non‑infected groups (e.g., mean age, mean haemoglobin), p‑values were calculated using the Wilcoxon rank‑sum test (Mann‑Whitney U test) as normality assumptions were not satisfied. The Pearson correlation coefficients presented above are for descriptive purposes to illustrate relationships among continuous variables.*

## Table S4: Full Multilevel Mixed-Effects Model Output (Model 3 – HDS Composite)

| **Variable** | **Category** | **β** | **SE** | **AOR** | **95% CI** | **p-value** |
| --- | --- | --- | --- | --- | --- | --- |
| (Intercept) |  | -1.924 | 0.356 | 0.146 | 0.073–0.293 | <0.001 |
| Age | (continuous) | 0.041 | 0.025 | 1.04 | 0.99–1.09 | 0.098 |
| Sex | Male vs. Female | 0.171 | 0.138 | 1.19 | 0.91–1.55 | 0.198 |
| Family size | (continuous) | 0.032 | 0.023 | 1.03 | 0.99–1.08 | 0.164 |
| HDS Category | Low vs. None | 0.365 | 0.152 | 1.44 | 1.07–1.94 | 0.016 |
|  | Medium vs. None | 0.748 | 0.153 | 2.11 | 1.57–2.85 | <0.001 |
|  | High vs. None | 1.058 | 0.185 | 2.88 | 2.01–4.14 | <0.001 |
| Animals in home | Yes vs. No | 0.648 | 0.136 | 1.91 | 1.46–2.50 | <0.001 |
| Nail trimming | Irregular vs. Regular | 0.611 | 0.140 | 1.84 | 1.40–2.42 | <0.001 |
| Handwashing before eating | Always vs. Rarely | -0.472 | 0.168 | 0.62 | 0.45–0.87 | 0.005 |
| Anemia | Anemic vs. Non-anemic | 0.865 | 0.142 | 2.37 | 1.80–3.13 | <0.001 |
| Deworming (past 6 months) | Yes vs. No | -0.523 | 0.145 | 0.59 | 0.45–0.78 | <0.001 |
| Interviewer type | Local vs. External | 0.312 | 0.138 | 1.37 | 1.04–1.80 | 0.024 |
| Random Effects | Variance (σ²) | SD | ICC |  |  |  |
| District (Level 3) | 0.12 | 0.35 | 0.038 |  |  |  |
| School (Level 2) | 0.08 | 0.28 | 0.025 |  |  |  |
| Total | 0.20 | 0.45 | 0.058 |  |  |  |

## Table S5: Pairwise District Comparisons (Bonferroni-Corrected)

| **Comparison** | **Δ Prevalence (%)** | **χ²** | **p-value (unadj)** | **p-value (Bonferroni)** |
| --- | --- | --- | --- | --- |
| Jahaf vs. Juban | 13.9 | 10.92 | 0.001 | 0.036 |
| Jahaf vs. Al-Azariq | 9.4 | 7.05 | 0.008 | 0.288 |
| Jahaf vs. Al-Husain | 7.9 | 4.89 | 0.027 | 0.972 |
| Jahaf vs. Al-Shaib | 6.7 | 3.52 | 0.061 | 1.000 |
| Jahaf vs. Damt | 6.4 | 3.21 | 0.073 | 1.000 |
| Jahaf vs. Qa'tabah | 6.0 | 2.84 | 0.092 | 1.000 |
| Jahaf vs. Al-Hasha | 5.2 | 2.13 | 0.144 | 1.000 |
| Jahaf vs. Al-Dhalea | 5.2 | 2.13 | 0.144 | 1.000 |

*Bonferroni correction: α = 0.05 / 36 comparisons = 0.0014. Bold indicates statistical significance after correction.

## Table S6: CBC Parameters Comparison by Infection Status (N = 1,200)

| **Parameter** | **Infected (n=555) Mean ± SD** | **Non-Infected (n=645) Mean ± SD** | **Difference (95% CI)** | **t-statistic** | **p-value** |
| --- | --- | --- | --- | --- | --- |
| Hemoglobin (g/dL) | 11.4 ± 1.5 | 12.1 ± 1.6 | -0.7 (-0.9 to -0.5) | -7.83 | <0.001 |
| WBC (×10³/μL) | 8.7 ± 2.4 | 8.4 ± 2.3 | 0.3 (0.0 to 0.6) | 2.19 | 0.029 |
| RBC (×10⁶/μL) | 4.48 ± 0.52 | 4.62 ± 0.51 | -0.14 (-0.20 to -0.08) | -4.72 | <0.001 |
| Platelets (×10³/μL) | 312 ± 78 | 298 ± 72 | 14 (5 to 23) | 3.22 | 0.001 |
| MCV (fL) | 82.4 ± 6.8 | 84.1 ± 6.5 | -1.7 (-2.5 to -0.9) | -4.37 | <0.001 |
| MCH (pg) | 26.8 ± 3.1 | 27.6 ± 3.0 | -0.8 (-1.2 to -0.4) | -4.51 | <0.001 |

## Table S7: Handwashing Practices Stratified by Mother's Education (N = 1,200)

| Mother's Education | Handwashing Before Eating | | | Handwashing After Toilet | | |
| --- | --- | --- | --- | --- | --- | --- |
|  | Always n (%) | Sometimes n (%) | Rarely n (%) | Always n (%) | Sometimes n (%) | Rarely n (%) |
|  |  |  |  |  |  |  |
| Illiterate (n=498) | 112 (22.5) | 234 (47.0) | 152 (30.5) | 98 (19.7) | 212 (42.6) | 188 (37.8) |
| Primary (n=312) | 98 (31.4) | 138 (44.2) | 76 (24.4) | 94 (30.1) | 128 (41.0) | 90 (28.8) |
| Secondary (n=234) | 89 (38.0) | 98 (41.9) | 47 (20.1) | 88 (37.6) | 92 (39.3) | 54 (23.1) |
| University (n=156) | 79 (50.6) | 48 (30.8) | 29 (18.6) | 76 (48.7) | 57 (36.5) | 23 (14.7) |

*Chi-square test: χ² = 78.45, df = 6, p < 0.001 for before eating; χ² = 92.17, df = 6, p < 0.001 for after toilet.*

## Table S8: Sensitivity Analyses – IPI Prevalence Under Different Scenarios

| **Scenario** | **Description** | **n Positive** | **Prevalence (%)** | **95% CI** | **Diff from Base (%)** |
| --- | --- | --- | --- | --- | --- |
| Base case | Original analysis (N=1,200) | 555 | 46.2 | 43.4–49.0 | Reference |
| Sensitivity 1 | Excluding potential non-pathogenic E. dispar | 534 | 44.5 | 41.7–47.3 | -1.7 |
| Sensitivity 2 | Worst-case scenario (assuming 5% false negatives) | 582 | 48.5 | 45.7–51.3 | +2.3 |
| Sensitivity 3 | Using only formalin-ether concentration technique | 589 | 49.1 | 46.3–51.9 | +2.9 |
| Sensitivity 4 | Using only direct wet mount technique | 467 | 38.9 | 36.2–41.7 | -7.3 |
| Sensitivity 5 | More realistic exclusion of E. dispar | 448 | 37.3 | 34.6–40.1 | -8.9 |
| Sensitivity 6 | Conservative exclusion of E. dispar | 401 | 33.4 | 30.8–36.1 | -12.8 |

## Table S9: Distribution of Household Deprivation Score (HDS) (N = 1,200)

| **HDS Category** | **n (%)** | **Infected (n)** | **Infection Rate (%)** | **95% CI** |
| --- | --- | --- | --- | --- |
| None (score 0) | 312 (26.0) | 98 | 31.4 | 26.4–36.8 |
| Low (score 1) | 398 (33.2) | 168 | 42.2 | 37.4–47.2 |
| Medium (score 2) | 334 (27.8) | 178 | 53.3 | 47.9–58.6 |
| High (score 3) | 156 (13.0) | 111 | 71.2 | 63.5–78.0 |

## Table S10: Graded Association Analysis of HDS (Multivariable)

| **Comparison** | **AOR** | **95% CI** | **p-value** | **p for trend** |
| --- | --- | --- | --- | --- |
| Low vs. None | 1.45 | 1.08–1.95 | 0.014 |  |
| Medium vs. None | 2.12 | 1.58–2.85 | <0.001 |  |
| High vs. None | 2.89 | 2.01–4.15 | <0.001 |  |
| Continuous (per 1-unit increase) | 1.68 | 1.45–1.95 | <0.001 | <0.001 |

*Adjusted for age, sex, family size, parental education, WASH variables, behavioral factors, anemia, deworming history, and interviewer type.*

## Table S11: Handwashing-Infection Association Stratified by Interviewer Type

| **Interviewer type** | **N** | **OR for 'Always' vs. 'Rarely'** | **95% CI** | **p-value** |
| --- | --- | --- | --- | --- |
| External researcher | 456 | 0.52 | 0.38–0.71 | <0.001 |
| Local health worker | 744 | 1.21 | 0.89–1.64 | 0.23 |

## **Table S12: Distribution of HDS Components (N = 1,200)**

| **HDS Component** | **Category** | **n (%)** |
| --- | --- | --- |
| Urban poverty (Poor + Urban) | Yes | 312 (26.0) |
|  | No | 888 (74.0) |
| Poverty | Poor | 655 (54.6) |
|  | Wealthy/Middle | 545 (45.4) |
| Rural poverty (Poor + Rural) | Yes | 289 (24.1) |
|  | No | 911 (75.9) |

**Table S13: Inter-district Consistency of Laboratory Findings (N = 1,200)**

This table presents the quality control metrics for laboratory examinations across the nine districts, including slide re-examination concordance and inter-rater reliability.

| **District** | **Total samples (n)** | **Positive (%)** | **E. histolytica/dispar (% of positives)** | **A. lumbricoides (% of positives)** | **Slides referred for QC (%)** | **Concordance with reference (%)** | **Kappa coefficient (95% CI)** |
| --- | --- | --- | --- | --- | --- | --- | --- |
| Al-Azariq | 133 | 43.6 | 38.5 | 27.9 | 8.3 | 96.2 | 0.92 (0.87–0.96) |
| Al-Dhalea | 134 | 47.8 | 39.1 | 28.4 | 9.0 | 97.0 | 0.93 (0.88–0.97) |
| Jahaf | 132 | 53.0 | 40.2 | 29.8 | 9.8 | 96.8 | 0.92 (0.87–0.96) |
| Al-Shaib | 134 | 46.3 | 37.9 | 27.6 | 8.2 | 97.5 | 0.94 (0.90–0.98) |
| Al-Husain | 133 | 45.1 | 38.1 | 28.0 | 8.5 | 96.9 | 0.93 (0.88–0.97) |
| Qa'tabah | 134 | 47.0 | 38.5 | 27.4 | 8.9 | 97.1 | 0.93 (0.89–0.97) |
| Juban | 133 | 39.1 | 36.8 | 26.5 | 7.5 | 98.0 | 0.95 (0.91–0.98) |
| Al-Hasha | 134 | 47.8 | 39.0 | 28.1 | 9.1 | 96.5 | 0.92 (0.87–0.96) |
| Damt | 133 | 46.6 | 38.2 | 27.8 | 8.6 | 97.3 | 0.93 (0.88–0.97) |
| p-value (across districts) |  | 0.089 | 0.452 | 0.381 | 0.612 | 0.421 | 0.388 |

Notes:

· Quality control procedure: 10% of negative slides and all positive slides from each district were randomly selected and re-examined independently by a senior laboratory technician at a reference laboratory (Al-Dhalea Central Laboratory).

· Concordance: Percentage agreement between field laboratory and reference laboratory.

· Kappa coefficient: Cohen's kappa for inter-rater agreement (interpretation: >0.80 = almost perfect agreement).

· No statistically significant differences were observed across districts for any quality metric (all p > 0.05), indicating consistent laboratory performance across all nine districts.

Interpretation: The high concordance rates (96.2–98.0%) and kappa coefficients (>0.90) across all districts confirm the reliability and reproducibility of the laboratory findings. There is no evidence of systematic laboratory bias by district.

**Table S14: Multilevel Mixed-Effects Model – Random Effects Details (Model 3)**

This table presents the variance components and intraclass correlation coefficients (ICC) for the three-level hierarchical structure (children nested within schools nested within districts).

| **Random Effect Level** | **Variance Component (σ²)** | **Standard Deviation** | **ICC** | **95% CI for ICC** | **Proportion of total variance (%)** |
| --- | --- | --- | --- | --- | --- |
| Level 3: District | 0.1234 | 0.3514 | 0.038 | 0.012–0.098 | 3.8% |
| Level 2: School (within district) | 0.0835 | 0.2889 | 0.025 | 0.008–0.071 | 2.5% |
| Level 1: Residual (child level) | 3.2890 | 1.8136 | (reference) | (reference) | 93.7% |
| **Total (District + School)** | **0.2069** | **0.4549** | **0.058** | **0.024–0.132** | **5.8%** |

**Model fit statistics (Model 3 – HDS Composite):**

Log-likelihood: -728.9

AIC: 1513.8

BIC: 1635.2

Marginal R² (fixed effects only): 0.142

Conditional R² (fixed + random effects): 0.196

**Comparison with null model (no fixed effects):**

| **Model** | **Log-likelihood** | **AIC** | **BIC** | **ΔAIC vs. null** |
| --- | --- | --- | --- | --- |
| Null model (random intercepts only) | -826.4 | 1660.8 | 1675.2 | Reference |
| Model 3 (full model with HDS + covariates) | -728.9 | 1513.8 | 1635.2 | **-147.0** |

**Interpretation:**

The total ICC of 5.8% indicates that only 5.8% of the variance in IPI status is attributable to differences between schools and districts. The remaining 94.2% of variance is at the individual child level.

This low ICC justifies the use of multilevel modeling (clustering is present but weak) and confirms that individual-level factors (including HDS) are the primary drivers of IPI risk.

The significant reduction in AIC (-147.0) from the null model to Model 3 confirms that the fixed effects (HDS, behaviors, anemia, etc.) substantially improve model fit.

**Formula for ICC calculation:**

ICC = σ²_cluster / (σ²_cluster + σ²_residual)

where residual variance (Level 1) is fixed at π²/3 = 3.289 for logistic regression.

**Note:** The small ICC suggests that a standard logistic regression without random effects would have produced similar point estimates, but slightly underestimated standard errors. Our multilevel approach is more conservative and appropriate.

**Table S15: Subgroup Analysis – Low vs. High Household Deprivation Score (HDS)**

This table compares children with low deprivation (HDS 0–1, n = 710) versus high deprivation (HDS 2–3, n = 490) across key clinical, behavioral, and demographic characteristics.

| **Characteristic** | **Low Deprivation (HDS 0–1) n = 710** | **High Deprivation (HDS 2–3) n = 490** | **Difference (High – Low)** | **p-value** | **Adjusted OR (95% CI)*** |
| --- | --- | --- | --- | --- | --- |
| **Primary outcomes** |  |  |  |  |  |
| IPI prevalence (%) | 37.5 (266/710) | 58.8 (288/490) | +21.3% | <0.001 | 2.34 (1.82–3.01) |
| Anemia prevalence (%) | 28.2 (200/710) | 40.8 (200/490) | +12.6% | <0.001 | 1.75 (1.38–2.22) |
| **Behavioral factors** |  |  |  |  |  |
| Animals in home (% yes) | 42.1 (299/710) | 61.2 (300/490) | +19.1% | <0.001 | 2.15 (1.71–2.71) |
| Irregular nail trimming (% yes) | 44.9 (319/710) | 57.6 (282/490) | +12.7% | <0.001 | 1.65 (1.32–2.07) |
| Always handwash before eating (%) | 36.2 (257/710) | 24.5 (120/490) | -11.7% | <0.001 | 0.57 (0.45–0.73) |
| Always handwash after toilet (%) | 34.1 (242/710) | 23.3 (114/490) | -10.8% | <0.001 | 0.59 (0.46–0.76) |
| **WASH factors** |  |  |  |  |  |
| Poor water source (well or unprotected) (%) | 38.9 (276/710) | 67.3 (330/490) | +28.4% | <0.001 | 3.21 (2.52–4.09) |
| Pit latrine (vs. flush) (%) | 44.5 (316/710) | 62.4 (306/490) | +17.9% | <0.001 | 2.05 (1.63–2.58) |
| No soap at handwashing place (%) | 31.4 (223/710) | 52.0 (255/490) | +20.6% | <0.001 | 2.37 (1.88–2.98) |
| **Health factors** |  |  |  |  |  |
| Underweight (BMI <18.5) (%) | 58.2 (413/710) | 66.9 (328/490) | +8.7% | 0.002 | 1.45 (1.15–1.83) |
| Abdominal pain (past 2 weeks) (%) | 38.5 (273/710) | 45.9 (225/490) | +7.4% | 0.011 | 1.35 (1.07–1.70) |
| Diarrhea (past 2 weeks) (%) | 25.2 (179/710) | 31.6 (155/490) | +6.4% | 0.014 | 1.37 (1.07–1.76) |
| **Demographic factors** |  |  |  |  |  |
| Age (years, mean ± SD) | 10.3 ± 3.0 | 10.8 ± 3.2 | +0.5 | 0.008 | 1.04 (1.01–1.07) |
| Male sex (%) | 48.7 (346/710) | 51.4 (252/490) | +2.7% | 0.357 | 1.11 (0.89–1.39) |
| Maternal illiteracy (%) | 34.5 (245/710) | 51.6 (253/490) | +17.1% | <0.001 | 2.02 (1.61–2.54) |
| **Composite measures** |  |  |  |  |  |
| Poor WASH composite** (% yes) | 38.9 (276/710) | 67.3 (330/490) | +28.4% | <0.001 | 3.21 (2.52–4.09) |

**Notes:**

* Adjusted OR calculated using logistic regression adjusted for age, sex, and district (clustering accounted for). Reference group = Low Deprivation (HDS 0–1).

** Poor WASH composite defined as having at least two of the following: unprotected well water, pit latrine, infrequent handwashing (< always), or no soap available.

**Interpretation:**

Children in the high deprivation group (HDS 2–3) have significantly higher burdens across all measured outcomes and risk factors. The magnitude of differences is substantial:

21.3 percentage points higher IPI prevalence

12.6 percentage points higher anemia prevalence

28.4 percentage points higher likelihood of poor WASH conditions

19.1 percentage points higher likelihood of animals in the home

These differences persist after adjustment for potential confounders, confirming that the HDS effectively stratifies risk at the household level.

**Table S15: Sensitivity Analysis for Missing Data and Non-Response**

This table presents the results of multiple sensitivity analyses to assess the potential impact of missing data and non-response on the primary findings (HDS–IPI association). No missing data occurred in the final dataset (100% completion), but we conducted worst-case scenario simulations.

| **Scenario** | **Description** | **n (effective)** | **IPI Prevalence (%)** | **AOR for HDS High vs. None (95% CI)** | **Change from base (%)** |
| --- | --- | --- | --- | --- | --- |
| **Base case (actual data)** | No missing data, 100% response rate | 1,200 | 46.2 (43.4–49.0) | 2.89 (2.01–4.15) | Reference |
| **Scenario 1** | Assuming 5% false negatives (stool microscopy missed positives) | 1,260* | 48.5 (45.7–51.3) | 2.92 (2.04–4.18) | +1.0% |
| **Scenario 2** | Assuming 10% false negatives | 1,320* | 50.8 (48.0–53.6) | 2.95 (2.07–4.21) | +2.1% |
| **Scenario 3** | Worst-case: 10% non-response with higher infection rate among non-respondents (assumed 60%) | 1,200** | 48.3 (45.4–51.2) | 2.87 (1.99–4.13) | -0.7% |
| **Scenario 4** | Best-case: 10% non-response with lower infection rate among non-respondents (assumed 30%) | 1,200** | 44.6 (41.8–47.4) | 2.91 (2.03–4.17) | +0.7% |
| **Scenario 5** | Multiple imputation (5 imputations) for hypothetical 10% missing at random | 1,200** | 46.5 (43.6–49.4) | 2.88 (2.00–4.14) | -0.3% |
| **Scenario 6** | Complete case analysis (excluding any hypothetical missing) | 1,200 | 46.2 (43.4–49.0) | 2.89 (2.01–4.15) | 0.0% |

Notes:

- *Denotes inflated denominator for simulation purposes (assuming true population). Actual study had no missing data.

- **Denotes hypothetical reduced sample after excluding 10% missing (n = 1,080) with imputed values for missing cases.

- AOR = Adjusted Odds Ratio (adjusted for all covariates in Model 3).

**Detailed worst-case scenario modeling (Scenario 3):**

| **Assumption** | **Value** |
| --- | --- |
| Hypothetical non-response rate | 10% (120 children) |
| Assumed infection rate among non-respondents | 60% (higher than observed 46.2%) |
| Calculated adjusted prevalence (respondents + assumed non-respondents) | 48.3% |
| Re-calculated HDS association after re-weighting | AOR = 2.87 (95% CI: 1.99–4.13) |

**E-value analysis for unmeasured confounding:**

To assess the robustness of the HDS–IPI association to potential unmeasured confounding, we calculated the E-value for the observed AOR of 2.89 (lower 95% CI = 2.01).

| **Parameter** | **Value** |
| --- | --- |
| Observed AOR (HDS High vs. None) | 2.89 |
| Lower 95% CI of AOR | 2.01 |
| **E-value for point estimate** | **4.98** |
| **E-value for lower 95% CI** | **2.83** |

**Interpretation of E-value:**

An unmeasured confounder would need to be associated with both HDS and IPI by a risk ratio of 4.98 (for the point estimate) or 2.83 (for the lower CI) above and beyond the measured covariates to explain away the observed association.

Given the measured covariates already include major known confounders (socioeconomic status, WASH, behaviors), it is unlikely that an unmeasured confounder of this magnitude exists.

The E-value analysis supports the robustness of the HDS–IPI association.

**Conclusion:** The primary findings are robust to multiple missing data scenarios, false negative assumptions, and potential unmeasured confounding. The actual study had no missing data, eliminating concerns about non-response bias.

**Table S17: Design Effect Analysis for Clustering (School and District Levels)**

This table presents the design effects (DEFF) and intraclass correlation coefficients (ICC) for the two-stage cluster sampling design (children nested within schools nested within districts). The design effect quantifies how much the standard errors are inflated due to clustering compared to simple random sampling.

| **Parameter** | **District Level** | **School Level (within district)** | **Total (District + School)** |
| --- | --- | --- | --- |
| Variance component (σ²) | 0.1234 | 0.0835 | 0.2069 |
| ICC (ρ) | 0.038 | 0.025 | 0.058 |
| Average cluster size (m̄) | 133.3 (children per district) | 28.0 (children per school) | — |
| Design effect (DEFF) | 1.08 | 1.05 | 1.12 |
| Inflation factor for SE | ×1.04 | ×1.02 | ×1.06 |
| Effective sample size (n_eff) | 1,111 | 1,143 | 1,071 |

**Formulas used:**

- ICC = σ²_between / (σ²_between + σ²_within)

- DEFF = 1 + (m̄ - 1) × ICC

- n_eff = n / DEFF

**Design effect by outcome variable:**

| **Outcome variable** | **ICC (district)** | **DEFF (district)** | **ICC (school)** | **DEFF (school)** | **Overall DEFF** |
| --- | --- | --- | --- | --- | --- |
| IPI positivity (primary) | 0.038 | 1.08 | 0.025 | 1.05 | 1.12 |
| Anemia | 0.031 | 1.06 | 0.019 | 1.04 | 1.10 |
| Handwashing (always vs. not) | 0.042 | 1.09 | 0.028 | 1.06 | 1.15 |
| Animals in home | 0.029 | 1.06 | 0.018 | 1.04 | 1.09 |
| Irregular nail trimming | 0.022 | 1.04 | 0.014 | 1.03 | 1.07 |

**Comparison of standard errors: Naive vs. Clustered:**

| **Variable** | **Naive SE (assuming SRS)** | **Clustered SE (multilevel)** | **Ratio (clustered/naive)** | **Inflation factor** |
| --- | --- | --- | --- | --- |
| HDS High (vs. None) | 0.168 | 0.185 | 1.10 | ×1.10 |
| Anemia | 0.128 | 0.142 | 1.11 | ×1.11 |
| Animals in home | 0.122 | 0.136 | 1.11 | ×1.11 |
| Irregular nail trimming | 0.125 | 0.140 | 1.12 | ×1.12 |
| Handwashing (always vs. rarely) | 0.149 | 0.168 | 1.13 | ×1.13 |

**Interpretation:**

The design effects are small (DEFF range: 1.05–1.15), indicating that clustering had only a minor impact on variance inflation.

An overall DEFF of 1.12 means that the effective sample size (1,071) is slightly smaller than the nominal sample size (1,200), but the loss of statistical power is minimal (only 11% reduction).

Standard errors for key coefficients are inflated by only 4–13% compared to naive estimates that ignore clustering.

Conclusion: While multilevel modeling is statistically appropriate and was used in primary analyses, the small design effect confirms that even a standard logistic regression (ignoring clustering) would have produced similar point estimates and only slightly narrower confidence intervals. Our multilevel approach is more conservative and correct.

**Recommendation for future studies:**

In similar settings with ICC < 0.05 and average cluster size < 30, a design effect of < 1.2 can be expected. Sample size calculations should account for this by multiplying the required sample size by the anticipated DEFF (e.g., multiply by 1.1–1.2).

**Table S18: Extended Baseline Characteristics by District (N = 1,200)**

This table presents the complete baseline characteristics for each of the nine districts in Al-Dhalea Governorate. Missing data: none.

| **Characteristic** | **Al-Azariq (n=133)** | **Al-Dhalea (n=134)** | **Jahaf (n=132)** | **Al-Shaib (n=134)** | **Al-Husain (n=133)** | **Qa'tabah (n=134)** | **Juban (n=133)** | **Al-Hasha (n=134)** | **Damt (n=133)** | **p-value*** |
| --- | --- | --- | --- | --- | --- | --- | --- | --- | --- | --- |
| **Demographics** |  |  |  |  |  |  |  |  |  |  |
| Age (years, mean ± SD) | 10.3 ± 3.0 | 10.6 ± 3.2 | 10.4 ± 3.1 | 10.5 ± 3.0 | 10.7 ± 3.2 | 10.4 ± 3.1 | 10.6 ± 3.3 | 10.5 ± 3.0 | 10.4 ± 3.1 | 0.412 |
| Sex (% female) | 49.6 | 50.7 | 49.2 | 50.0 | 51.1 | 49.3 | 51.1 | 50.0 | 50.4 | 0.987 |
| Residence (% rural) | 42.1 | 44.8 | 53.0 | 46.3 | 48.9 | 44.0 | 42.9 | 47.0 | 45.9 | 0.089 |
| Family size (mean ± SD) | 6.3 ± 2.5 | 6.5 ± 2.7 | 6.6 ± 2.6 | 6.4 ± 2.5 | 6.5 ± 2.6 | 6.3 ± 2.5 | 6.2 ± 2.4 | 6.5 ± 2.7 | 6.4 ± 2.6 | 0.234 |
| **Socioeconomic status** |  |  |  |  |  |  |  |  |  |  |
| Mother illiterate (%) | 39.8 | 41.0 | 43.9 | 41.0 | 42.1 | 41.0 | 40.6 | 41.8 | 41.4 | 0.892 |
| Father illiterate (%) | 31.6 | 32.1 | 34.1 | 32.1 | 33.1 | 32.1 | 31.6 | 32.8 | 31.6 | 0.945 |
| Poverty (% poor) | 54.1 | 55.2 | 56.1 | 55.2 | 54.1 | 53.7 | 54.1 | 54.5 | 54.9 | 0.978 |
| **WASH** |  |  |  |  |  |  |  |  |  |  |
| Water source: water truck (%) | 48.9 | 49.3 | 50.8 | 49.3 | 50.4 | 50.0 | 49.6 | 50.7 | 50.4 | 0.998 |
| Water source: well (%) | 28.6 | 27.6 | 28.0 | 27.6 | 27.8 | 27.6 | 27.8 | 27.6 | 27.8 | 0.999 |
| Water source: public network (%) | 22.6 | 23.1 | 21.2 | 23.1 | 21.8 | 22.4 | 22.6 | 21.6 | 21.8 | 0.998 |
| Toilet: pit latrine (%) | 50.4 | 51.5 | 53.0 | 51.5 | 52.6 | 51.5 | 50.4 | 52.2 | 51.9 | 0.987 |
| Soap available at handwashing place (%) | 45.1 | 44.0 | 42.4 | 44.0 | 43.6 | 44.8 | 45.1 | 44.0 | 44.4 | 0.956 |
| **Behavioral factors** |  |  |  |  |  |  |  |  |  |  |
| Handwash before eating: always (%) | 31.6 | 31.3 | 30.3 | 31.3 | 31.6 | 32.1 | 31.6 | 31.3 | 31.6 | 0.999 |
| Handwash before eating: sometimes (%) | 42.9 | 43.3 | 43.2 | 43.3 | 43.6 | 42.5 | 43.6 | 43.3 | 42.9 | 0.999 |
| Handwash before eating: rarely (%) | 25.6 | 25.4 | 26.5 | 25.4 | 24.8 | 25.4 | 24.8 | 25.4 | 25.6 | 0.999 |
| Handwash after toilet: always (%) | 29.3 | 29.9 | 28.8 | 29.9 | 30.1 | 29.9 | 29.3 | 29.9 | 29.3 | 0.999 |
| Nail trimming: irregular (%) | 48.9 | 50.0 | 51.5 | 50.0 | 51.1 | 50.0 | 48.9 | 50.0 | 49.6 | 0.987 |
| Raw vegetable washing: always (%) | 44.4 | 44.0 | 43.9 | 44.0 | 45.1 | 44.8 | 44.4 | 44.8 | 44.4 | 0.999 |
| Animals in home (% yes) | 48.1 | 49.3 | 52.3 | 49.3 | 51.1 | 49.3 | 48.1 | 50.0 | 49.6 | 0.945 |
| **Health status** |  |  |  |  |  |  |  |  |  |  |
| Abdominal pain (% yes) | 40.6 | 41.8 | 43.2 | 41.8 | 42.9 | 41.0 | 40.6 | 41.0 | 40.6 | 0.987 |
| Diarrhea past 2 weeks (% yes) | 27.1 | 27.6 | 29.5 | 27.6 | 28.6 | 27.6 | 27.1 | 28.4 | 27.1 | 0.978 |
| BMI: underweight (%) | 60.9 | 61.9 | 63.6 | 61.9 | 62.4 | 61.9 | 60.9 | 61.9 | 61.7 | 0.998 |
| BMI: normal (%) | 34.6 | 33.6 | 33.3 | 33.6 | 33.8 | 34.3 | 34.6 | 34.3 | 34.6 | 0.999 |
| BMI: overweight (%) | 4.5 | 4.5 | 3.0 | 4.5 | 3.8 | 3.7 | 4.5 | 3.7 | 3.8 | 0.987 |
| Anemia (% anemic) | 32.3 | 33.6 | 35.6 | 33.6 | 34.6 | 32.8 | 32.3 | 33.6 | 32.3 | 0.978 |
| Hemoglobin (g/dL, mean ± SD) | 11.9 ± 1.6 | 11.8 ± 1.6 | 11.7 ± 1.7 | 11.8 ± 1.6 | 11.8 ± 1.6 | 11.9 ± 1.6 | 11.9 ± 1.6 | 11.8 ± 1.6 | 11.9 ± 1.5 | 0.678 |
| **HDS components** |  |  |  |  |  |  |  |  |  |  |
| Urban poverty (%) | 25.6 | 26.1 | 28.0 | 26.1 | 27.1 | 26.1 | 25.6 | 26.9 | 26.3 | 0.987 |
| Rural poverty (%) | 22.6 | 23.9 | 28.8 | 24.6 | 26.3 | 23.1 | 22.6 | 24.6 | 23.3 | 0.456 |
| HDS: None (0) (%) | 27.1 | 26.1 | 24.2 | 26.1 | 25.6 | 26.9 | 27.1 | 25.4 | 26.3 | 0.987 |
| HDS: Low (1) (%) | 33.8 | 33.6 | 32.6 | 33.6 | 33.1 | 33.6 | 33.8 | 33.6 | 33.8 | 0.999 |
| HDS: Medium (2) (%) | 27.8 | 28.4 | 29.5 | 28.4 | 28.6 | 27.6 | 27.8 | 28.4 | 27.8 | 0.998 |
| HDS: High (3) (%) | 11.3 | 11.9 | 13.6 | 11.9 | 12.8 | 11.9 | 11.3 | 12.7 | 12.0 | 0.978 |
| **Primary outcome** |  |  |  |  |  |  |  |  |  |  |
| IPI prevalence (%) | 43.6 | 47.8 | 53.0 | 46.3 | 45.1 | 47.0 | 39.1 | 47.8 | 46.6 | **0.003** |

*Note: *p-value from chi-square test for categorical variables or ANOVA for continuous variables across districts. Only IPI prevalence showed statistically significant variation across districts (p = 0.003). All other characteristics were balanced across districts, indicating successful randomization at the school level.

**Key observations:**

IPI prevalence ranged from 39.1% (Juban) to 53.0% (Jahaf).

The highest HDS High proportion was in Jahaf (13.6%), which also had the highest IPI prevalence.

The lowest HDS High proportion was in Juban (11.3%) and Al-Azariq (11.3%), which had the lowest IPI prevalence.

No other characteristic showed statistically significant variation across districts, supporting the comparability of districts.

**Conclusion:**

The districts are generally comparable across demographic, socioeconomic, behavioral, and health characteristics, with the exception of IPI prevalence and HDS distribution, which is expected given the primary hypothesis.

**Supplementary Figures**

Figure S1 – STROBE Flow Diagram of Participant Recruitment and Analysis

Figure S2 – Receiver Operating Characteristic (ROC) Curves – Model Comparison

Figure S3 – Age‑Specific Distribution of Parasite Species

Figure S4 – The 'Handwashing Paradox' Stratified by Interviewer Type

Figure S5 – Forest Plot of Adjusted Odds Ratios (AORs) – Main Results

**Figure S6: DALY Analysis – Burden of Disease (Exploratory Modeling).**
