# Supplementary material for: Household deprivation score demonstrates graded association with intestinal parasitic infections among schoolchildren in a conflict-affected setting: a cross-sectional study
Source: Front Public Health. 2026 Jul 8;14:1868011. doi: 10.3389/fpubh.2026.1868011 (PMC13388386; doi:10.3389/fpubh.2026.1868011)
Supplement: Supplementary file 4 [file Supplementary_file_4.pdf]

بسم الله الرحمن الرحيم

الجمهورية اليمنية
وزارة الصحة العامة والسكان
مكتب الصحة العامة والسكان
محافظة الضالع

التاريخ: ....
الموافق: ....
المرفقات: ....

Ref. No.: MoPHP/AD/EC/2025-028
Date: 15 March 2025

ETHICAL APPROVAL CERTIFICATE

To: Dr. Naif Taleb Ali
Principal Investigator
University of Science and Technology, Aden, Yemen

Protocol Title:
A Multi-District Investigation of Intestinal Parasitic Infections Among Schoolchildren in Rural Yemen: Integrating Clinical, Behavioral, and Machine Learning Analyses

The Ethical Committee for Health Research has reviewed the above-mentioned protocol and hereby grants full ethical approval for its conduct in all nine districts of Al-Dhalea Governorate, Yemen.

Approval Validity:
15 March 2025 – 15 September 2026

Conditions:

1. Use approved informed consent forms
2. Submit progress reports every 6 months
3. Report any serious adverse events immediately
4. Maintain participant confidentiality

Chairperson

____

Dr. Ayad Saleh Abdullah
Chairperson, Ethical Committee for Health Research
Ministry of Public Health and Population
Al-Dhalea Governorate Office

الجمهورية اليمنية
وزارة الصحة العامة والسكان
مكتب الصحة العامة
محافظة الضالع
