# Supplementary material for: Household deprivation score demonstrates graded association with intestinal parasitic infections among schoolchildren in a conflict-affected setting: a cross-sectional study
Source: Front Public Health. 2026 Jul 8;14:1868011. doi: 10.3389/fpubh.2026.1868011 (PMC13388386; doi:10.3389/fpubh.2026.1868011)
Supplement: Supplementary file 5 [file Supplementary_file_5.DOCX]

File S5: Informed Consent Forms

# S5.1: Parent/Guardian Informed Consent Form (English Version)

**Study Title:** Household Deprivation Score is a Scalable Screening Tool for Intestinal Parasitic Infections Among Schoolchildren in a Conflict-Affected Setting

**Principal Investigator:** Dr. Naif Taleb Ali

**Institution:** University of Science and Technology, Aden, Yemen

**Contact:** +967-772571390 | n.taleb@ust.edu

## Part 1: Information Sheet

### Introduction

We invite your child to participate in a research study about intestinal parasites (worms and other germs that live in the intestines). Before you decide, it is important to understand why the research is being done and what it will involve.

### Purpose of the Study

To determine how common intestinal parasites are among schoolchildren in Al-Dhalea Governorate, what factors increase the risk of infection, and how these infections affect children's health.

### Why has my child been chosen?

Your child has been chosen because they are between 5–15 years old and attend a school in one of the nine districts of Al-Dhalea Governorate. Approximately 1,200 children will participate.

### What will happen if my child takes part?

1. 1. Questionnaire: We will ask you questions about your household, water and sanitation, and your child's habits (20–25 minutes).
2. 2. Stool sample: Your child will provide a small stool sample (about the size of a grape) to be tested for intestinal parasites.
3. 3. Blood sample: A small blood sample (about 1 mL) will be taken from your child's fingertip to test for anemia.

### Are there any benefits?

Yes. If your child has intestinal parasites or anemia, they will receive free treatment according to Yemeni national health guidelines.

### Are there any risks?

The finger-prick may cause mild, temporary discomfort (like a small pinch). No other known risks.

### Will the information be kept confidential?

Yes. All information will be kept strictly confidential. Your child's name will not appear in any report or publication.

### Does my child have to take part?

No. Participation is completely voluntary. You may withdraw your child at any time without giving a reason and without any penalty.

### What if I have questions?

Contact Dr. Naif Taleb Ali at +967-772571390 or n.taleb@ust.edu.

## Part 2: Consent Statement

I have read the above information (or it has been read to me). I have had the opportunity to ask questions, and any questions I have asked have been answered to my satisfaction.

I understand that:

- My child's participation is voluntary
- I can withdraw my child at any time without giving a reason
- My child's information will be kept confidential
- My child will receive free treatment if infections are found

Please tick the boxes to confirm:

- ☐ I have read and understood the information sheet.
- ☐ I understand that participation is voluntary and that I can withdraw at any time.
- ☐ I understand that my child's information will be kept confidential.
- ☐ I agree to my child providing a stool sample for testing.
- ☐ I agree to my child providing a blood sample (finger-prick) for testing.
- ☐ I agree to be contacted about my child's test results.
- ☐ I agree for my child to receive free treatment if needed.

Parent/Guardian Name (printed): ______________________________

Parent/Guardian Signature: ______________________________

Date: ____ / ____ / ______

Relationship to Child: ______________________________

Child's Name (printed): ______________________________

Child's Date of Birth: ____ / ____ / ______

## Part 3: Researcher Declaration

I have explained the study to the parent/guardian to the best of my ability. I confirm that they have understood the information and have given free and informed consent.

Researcher Name (printed): ______________________________

Researcher Signature: ______________________________

Date: ____ / ____ / ______

# S5.2: Child Assent Form (English Version)

**Study Title:** Intestinal Parasites in Children

Hello! We are doing a study to learn about germs that can live in children's tummies. We would like to invite you to be part of this study.

### What will happen?

- We will ask your parent some questions about your home and your habits.
- You will collect a small poop sample in a clean container.
- We will take a tiny drop of blood from your fingertip (it feels like a small pinch).

### Are there any good things?

- If we find any bad germs, we will give you medicine to make them go away.
- You will learn how to stay healthy.

### Are there any bad things?

- The finger-prick might feel like a small pinch, but it goes away quickly.

### Do you have to say yes?

No. It is your choice. You can say no, and that's okay. No one will be upset with you. You can also change your mind later.

### Do you have questions?

You can ask us any questions you have, now or later.

Please tick the box:

- ☐ I understand what will happen in this study.
- ☐ I agree to take part in this study.

Child's Name (printed): ______________________________

Child's Signature (or mark): ______________________________

Date: ____ / ____ / ______

### Researcher's Statement:

I have explained the study to the child to the best of my ability. The child appeared to understand and freely agreed to participate.

Researcher Name (printed): ______________________________

Researcher Signature: ______________________________

Date: ____ / ____ / ______

**S5.1: Parent/Guardian Informed Consent Form (Arabic Version)**

**نموذج موافقة ولي/الوصي على المشاركة في البحث (النسخة العربية)**

__________________________________________________

**عنوان الدراسة: درجة الحرمان الأسري كأداة فحص قابلة للتطوير للعدوى الطفيلية المعوية بين أطفال المدارس في بيئة متأثرة بالنزاع**

الباحث الرئيسي: د. نايف طالب علي

الجهة: جامعة العلوم والتكنولوجيا، عدن، اليمن

للتواصل: +967-772571390 | n.taleb@ust.edu

__________________________________________________

**الجزء الأول: نشرة معلوماتية للأسرة**

**مقدمة**

نحن بدعوة طفلك/طفلتك للمشاركة في دراسة بحثية حول الطفيليات المعوية (الدود والجراثيم الأخرى التي تعيش في الأمعاء). قبل أن تقرر، من المهم أن تفهم لماذا يتم إجراء هذا البحث وما الذي سيشمله.

**الغرض من الدراسة**

تحديد مدى انتشار الطفيليات المعوية بين أطفال المدارس في محافظة الضالع، وما هي العوامل التي تزيد من خطر الإصابة، وكيف تؤثر هذه العدوى على صحة الأطفال.

**لماذا تم اختيار طفلي؟**

تم اختيار طفلك لأنه يتراوح عمره بين 5–15 سنة ويلتحق بمدرسة في إحدى مديريات محافظة الضالع التسع. سوف يشارك في هذه الدراسة ما يقارب 1,200 طفل.

**ماذا سيحدث إذا شارك طفلي؟**

1. استبيان: سنطرح عليك أسئلة حول أسرتك، والمياه والصرف الصحي، وعادات طفلك (20–25 دقيقة).

2. عينة براز: سيعطي طفلك عينة براز صغيرة (بحجم حبة العنب تقريباً) لفحصها بحثاً عن الطفيليات المعوية.

3. عينة دم: سيتم أخذ عينة دم صغيرة (حوالي 1 مل) من إصبع طفلك لفحص نسبة الهيموغلوبين (فحص فقر الدم).

**هل هناك أي فوائد؟**

نعم. إذا تبين أن طفلك مصاب بطفيليات معوية أو فقر دم، فسيحصل على علاج مجاني وفقاً للإرشادات الصحية الوطنية اليمنية.

**هل هناك أي مخاطر؟**

قد يسبب وخز الإصبع انزعاجاً بسيطاً ومؤقتاً (مثل قرصة صغيرة). لا توجد مخاطر أخرى معروفة.

**هل ستظل المعلومات سرية؟**

نعم. جميع المعلومات ستبقى سرية تامة. لن يظهر اسم طفلك في أي تقرير أو منشور.

**هل يجب على طفلي المشاركة؟**

لا. المشاركة تطوعية تماماً. يمكنك سحب طفلك في أي وقت بدون ذكر سبب وبدون أي عقوبة.

**ماذا لو كان لدي أسئلة؟**

يمكنك التواصل مع الدكتور نايف طالب علي على الرقم +967-772571390 أو البريد الإلكتروني n.taleb@ust.edu.

__________________________________________________

**الجزء الثاني: إقرار بالموافقة**

أنا، ولي/وصي الطفل، أقر بما يلي:

· لقد قرأت المعلومات أعلاه (أو تمت قراءتها لي).

· أتيحت لي الفرصة لطرح الأسئلة، وتمت الإجابة على جميع أسئلتي بشكل مرضٍ.

· أنا أفهم أن مشاركة طفلي تطوعية، ويمكنني سحبه في أي وقت.

· أنا أفهم أن معلومات طفلي ستبقى سرية.

· أنا أفهم أن طفلي سيحصل على علاج مجاني إذا تم العثور على أي عدوى.

**يرجى وضع علامة (✓) في المربعات المناسبة لتأكيد موافقتك:**

· ☐ لقد قرأت وفهمت نشرة المعلومات.

· ☐ أنا أفهم أن المشاركة تطوعية وأنه يمكنني سحب طفلي في أي وقت.

· ☐ أوافق على أن تبقى معلومات طفلي سرية.

· ☐ أوافق على تقديم طفلي عينة براز للفحص.

· ☐ أوافق على تقديم طفلي عينة دم (وخز الإصبع) للفحص.

· ☐ أوافق على أن يتم الاتصال بي لإبلاغي بنتائج فحوصات طفلي.

· ☐ أوافق على أن يتلقى طفلي العلاج المجاني إذا لزم الأمر.

__________________________________________________

اسم ولي الأمر / الوصي (كتابة): _________________________________

توقيع ولي الأمر / الوصي: _________________________________

التاريخ: ____ / ____ / ______

صلة القرابة بالطفل: _________________________________

اسم الطفل (كتابة): _________________________________

تاريخ ميلاد الطفل: ____ / ____ / ______

__________________________________________________

**الجزء الثالث: إقرار الباحث**

أنا، الباحث الموقع أدناه، أقر بما يلي:

· لقد شرحت الدراسة لولي/وصي الطفل بأفضل ما لدي من قدرة.

· أؤكد أن ولي/وصي الطفل قد فهم المعلومات المقدمة وقدم موافقته الحرة والمستنيرة.

اسم الباحث (كتابة): _________________________________

توقيع الباحث: _________________________________

التاريخ: ____ / ____ / ______

__________________________________________________

**S5.2: Child Assent Form (Arabic Version)**

**نموذج موافقة الطفل (النسخة العربية)**

__________________________________________________

**عنوان الدراسة: الطفيليات المعوية عند الأطفال**

**مرحباً!**

نحن نقوم بدراسة لمعرفة الجراثيم التي يمكن أن تعيش في بطون الأطفال. نود أن ندعوك لتكون جزءاً من هذه الدراسة.

**ماذا سيحدث؟**

· سنسأل ولي أمرك بعض الأسئلة حول منزلك وعاداتك.

· ستقوم بجمع عينة براز في وعاء نظيف.

· سنأخذ قطرة دم صغيرة جداً من طرف إصبعك (ستشعر وكأنها قرصة صغيرة).

**هل هناك أي أشياء جيدة؟**

· إذا وجدنا أي جراثيم ضارة، فسنعطيك دواءً لتجعلها تختفي.

· سوف تتعلم كيف تحافظ على صحتك.

**هل هناك أي أشياء سيئة؟**

· قد تشعر بوخز الإصبع كقرصة صغيرة، لكنها تختفي بسرعة.

**هل يجب أن توافق؟**

لا. هذا اختيارك. يمكنك أن تقول 'لا'، ولا بأس بذلك. لن ينزعج منك أحد. يمكنك أيضاً تغيير رأيك لاحقاً.

**هل لديك أسئلة؟**

يمكنك أن تسألنا أي سؤال لديك، الآن أو في أي وقت لاحق.

__________________________________________________

**يرجى وضع علامة (✓) في المربع:**

· ☐ أنا أفهم ما سيحدث في هذه الدراسة.

· ☐ أوافق على المشاركة في هذه الدراسة.

__________________________________________________

اسم الطفل (كتابة): _________________________________

توقيع الطفل (أو بصمة الإبهام): _________________________________

التاريخ: ____ / ____ / ______

__________________________________________________

**إقرار الباحث:**

أنا، الباحث الموقع أدناه، أقر بما يلي:

· لقد شرحت الدراسة للطفل بأفضل ما لدي من قدرة.

· يبدو أن الطفل قد فهم الشرح ووافق بحرية على المشاركة.

اسم الباحث (كتابة): _________________________________

توقيع الباحث: _________________________________

التاريخ: ____ / ____ / ______
