# Supplementary material for: Household deprivation score demonstrates graded association with intestinal parasitic infections among schoolchildren in a conflict-affected setting: a cross-sectional study
Source: Front Public Health. 2026 Jul 8;14:1868011. doi: 10.3389/fpubh.2026.1868011 (PMC13388386; doi:10.3389/fpubh.2026.1868011)
Supplement: Supplementary file 6 [file Supplementary_file_6.DOCX]

# File S6: STUDY QUESTIONNAIRE

**Study Title:** Household Deprivation Score is a Scalable Screening Tool for Intestinal Parasitic Infections Among Schoolchildren in a Conflict-Affected Setting

**Questionnaire ID:** _______________

## Section A: Demographic Information

**A1.** Student ID: _______________

**A2.** Age: _______ years

**A3.** Sex: □ Female □ Male

**A4.** District:

□ Al-Azariq □ Al-Dhalea □ Jahaf □ Al-Shaib □ Al-Husain

□ Qa'tabah □ Juban □ Al-Hasha □ Damt

**A5.** School Name: _______________

**A6.** Residence: □ Urban □ Rural

**A7.** Family size: _______ persons

## Section B: Socioeconomic Status

**B1.** Mother's Education:

□ Illiterate □ Primary □ Secondary □ University

**B2.** Father's Education:

□ Illiterate □ Primary □ Secondary □ University

**B3.** Household asset ownership (tick all that apply):

□ Refrigerator □ Television □ Washing machine □ Car

□ Motorcycle □ Bicycle □ Smartphone □ Computer/laptop □ Livestock

**B4.** Wealth classification (calculated):

□ Wealthy (6+ items) □ Middle (3–5 items) □ Poor (0–2 items)

## Section C: Water, Sanitation, and Hygiene (WASH)

**C1.** Primary source of drinking water:

□ Water truck □ Well □ Public network □ Bottled water □ Other: ______

**C2.** Type of toilet facility:

□ Flush toilet □ Pit latrine □ No toilet (open defecation) □ Other: ______

**C3.** Frequency of handwashing BEFORE eating:

□ Always □ Sometimes □ Rarely □ Never

**C4.** Frequency of handwashing AFTER using toilet:

□ Always □ Sometimes □ Rarely □ Never

**C5.** Is soap available at handwashing places? □ Yes □ No

## Section D: Behavioral Factors

**D1.** Nail trimming habits (for the child):

□ Regular (weekly or more) □ Irregular (less than weekly) □ Never trimmed

**D2.** Does the child bite their nails? □ Yes □ No

**D3.** Frequency of washing raw vegetables before eating:

□ Always □ Sometimes □ Never

**D4.** Does the child wear shoes regularly when outside?

□ Always □ Sometimes □ Rarely □ Never

**D5.** Does the child play in soil or sand?

□ Yes (daily) □ Yes (sometimes) □ No

## Section E: Household Environment

**E1.** Are animals kept inside the home or in the living area? □ Yes □ No

**E2.** If yes, what type? (tick all that apply)

□ Goats/sheep □ Cattle □ Poultry □ Cats □ Dogs □ Other: ______

**E3.** Are animal feces removed daily from the living area?

□ Yes □ No □ Not applicable

**E4.** Type of floor in the child's sleeping area:

□ Cement/tiles □ Earth/mud □ Wood □ Other: ______

## Section F: Health Status (Past 2 Weeks)

**F1.** Has the child experienced abdominal pain? □ Yes □ No

**F2.** Has the child experienced diarrhea (≥3 loose stools/day)? □ Yes □ No

**F3.** Has the child experienced nausea or vomiting? □ Yes □ No

**F4.** Has the child experienced loss of appetite? □ Yes □ No

**F5.** Has the child experienced weight loss in the past 3 months? □ Yes □ No

**F6.** Has the child been treated for intestinal parasites in the past 6 months? □ Yes □ No □ Don't know

**F7.** If yes, what medication? _______________

## Section G: Anthropometric Measurements

**G1.** Height: _______ cm (_______ m)

**G2.** Weight: _______ kg

**G3.** BMI: _______ kg/m²

**G4.** BMI Category:

□ Underweight (BMI for age <18.5) □ Normal (18.5–24.9) □ Overweight (≥25)

## Section H: Laboratory Results

**H1.** Hemoglobin: _______ g/dL □ Anemic □ Non-anemic

**H2.** WBC: _______ ×10³/μL

**H3.** RBC: _______ ×10⁶/μL

**H4.** Platelets: _______ ×10³/μL

**H5.** MCV: _______ fL

**H6.** MCH: _______ pg

**H7.** Stool examination (direct wet mount): □ Negative □ Positive (specify): _______

**H8.** Stool examination (formalin-ether concentration): □ Negative □ Positive (specify): _______

**H9.** Final infection status: □ Negative □ Positive

**H10.** Parasite type (if positive):

□ *Entamoeba histolytica/dispar* complex

□ *Giardia lamblia*

□ *Ascaris lumbricoides*

□ *Hymenolepis nana*

□ Hookworms

□ Other: _______

**H11.** Co-infection: □ Single □ Co-infection (species): _______

## Section I: Data Collection Information

**I1.** Data Collector Name/ID: _______________

**I2.** Date of Data Collection: ____ / ____ / ______

**I3.** Time of Sample Collection: ______ : ______ (24h)

**I4.** Quality Control Checked By: _______________
