# Supplementary material for: Household deprivation score demonstrates graded association with intestinal parasitic infections among schoolchildren in a conflict-affected setting: a cross-sectional study
Source: Front Public Health. 2026 Jul 8;14:1868011. doi: 10.3389/fpubh.2026.1868011 (PMC13388386; doi:10.3389/fpubh.2026.1868011)
Supplement: Supplementary file 8 [file Supplementary_file_8.DOCX]

File S8: Extended Sensitivity Analyses

# Important note for all tables in this file: All associations reported are statistical correlations derived from a cross-sectional study. Causal inference is not possible. The term "associated" is used descriptively.

# Table S8A: Sensitivity Analysis for HDS Using Non-Overlapping Definitions (Primary Sensitivity Analysis)

| Scenario | Variable Definition | AOR | 95% CI | p-value |
| --- | --- | --- | --- | --- |
| Primary (Urban Poverty) | Poor + Urban | 1.78 | 1.32–2.41 | <0.001 |
| Alternative urban poverty (non-overlapping) | Poor + Urban + Family size >6 | 1.68 | 1.23–2.29 | 0.001 |
| Alternative rural poverty (non-overlapping) | Poor + Rural + No school within 2 km | 1.91 | 1.38–2.64 | <0.001 |
| HDS as continuous | HDS Score (0–3) per 1-unit increase | 1.68 | 1.45–1.95 | <0.001 |
| Non-overlapping HDS (any deprivation) | Poor OR Urban-poor OR Rural-poor (mutually exclusive categories) | 2.76 | 2.01–3.78 | <0.001 |

Interpretation: All alternative non-overlapping definitions yielded effect sizes similar to the primary analysis (AOR range: 1.68–2.76), suggesting that the findings are robust to these alternative specifications. The non-overlapping definitions are methodologically superior because they avoid double-counting poverty, but the overlapping HDS (primary analysis) is presented for simplicity in field use (File S10).

# Table S8B: Sensitivity Analysis for Entamoeba Species Differentiation

| Assumption | Prevalence of E. histolytica/dispar complex (microscopy) | Adjusted Prevalence of Pathogenic E. histolytica | Change from Base |
| --- | --- | --- | --- |
| Base case (microscopy only) | 17.8% (214/1,200) | Not distinguished | Reference |
| Assuming 50% of Entamoeba are E. dispar | 17.8% | 8.9% (107/1,200) | -20% |
| Assuming 60% of Entamoeba are E. dispar | 17.8% | 7.1% (85/1,200) | -26% |
| Assuming 70% of Entamoeba are E. dispar | 17.8% | 5.3% (64/1,200) | -33% |
| Assuming 80% of Entamoeba are E. dispar | 17.8% | 3.6% (43/1,200) | -40% |
| Assuming 90% of Entamoeba are E. dispar | 17.8% | 1.8% (21/1,200) | -47% |

Note: Regional molecular epidemiology studies suggest that 70–80% of microscopy-positive Entamoeba in the Middle East may be non-pathogenic E. dispar, with some studies reporting up to 90%. These figures are estimates; molecular confirmation (PCR) in this specific population is urgently needed.

Interpretation for Table S8B (Entamoeba Species Differentiation):

These adjustments assume that 70–80% of microscopy-positive Entamoeba cases are non-pathogenic E. dispar, based on regional molecular epidemiology studies from the Middle East [Fotedar et al., 2007]. These are estimates; molecular confirmation (PCR) is needed to determine the true prevalence of pathogenic E. histolytica in this population. The wide range (50–90% non-pathogenic) reflects uncertainty in the literature; 70–80% represents the most plausible range for this setting, but local molecular data are urgently needed.

**Table S8C: Species-Stratified Analysis — Prevalence of Pathogenic Infections by HDS Category (Excluding Entamoeba Complex)**

| **HDS Category** | **N** | **Pathogenic Infections (n)** | **Prevalence (%) (95% CI)** |
| --- | --- | --- | --- |
| None (0) | 312 | 78 | 25.0 (20.4-30.1) |
| Low (1) | 287 | 82 | 28.6 (23.6-34.0) |
| Medium (2) | 389 | 135 | 34.7 (30.1-39.6) |
| High (3) | 212 | 80 | 37.7 (31.2-44.7) |

Note: Pathogenic infections defined as: Ascaris lumbricoides, Giardia lamblia, Hymenolepis nana, and hookworms. Entamoeba histolytica/dispar complex cases (n=214) were excluded; among these, 156 (29.4% of all positive participants) were Entamoeba-only cases potentially representing non-pathogenic E. dispar.

**Table S8D: Species-Stratified Analysis — Multilevel Model Results (Pathogenic Infections Only)**

| Comparison | AOR (95% CI) | p-value | Attenuation vs. Primary Analysis |
| --- | --- | --- | --- |
| Low (1) vs. | 1.31 (0.94-1.83) | 0.108 | 11.5% |
| None (0) |  |  |  |
| Medium (2) vs. | 1.76 (1.28-2.42) | < 0.001 | 19.3% |
| None (0) |  |  |  |
| High (3) vs. | 2.18 (1.47-3.23) | < 0.001 | 26.1% |
| None (0) |  |  |  |
| p for trend | — | < 0.001 | — |

Model fit: AIC = 1342.7, BIC = 1373.5

Interpretation: The graded association between HDS and pathogenic infections remains statistically significant for HDS ≥ 2 but is substantially attenuated compared to the primary analysis (which included Entamoeba cases). The loss of statistical significance for the low deprivation category (HDS = 1) and the 26.1% attenuation for the high deprivation category suggest that part of the observed HDS-IPI association is driven by non-pathogenic Entamoeba carriage. Molecular confirmation (PCR) is needed to distinguish pathogenic E. histolytica from non-pathogenic E. dispar.

# Table S8E: Sensitivity Analysis for Missing Data (Worst-Case Scenario)

| Missing Data Assumption | n Positive | Prevalence (%) | 95% CI |
| --- | --- | --- | --- |
| Base case (no missing) | 555 | 46.2 | 43.4–49.0 |
| Assuming 5% false negatives | 582 | 48.5 | 45.7–51.3 |
| Assuming 10% false negatives | 610 | 50.8 | 48.0–53.6 |

# Table S8F: Sensitivity Analysis for Interviewer Type (Handwashing Paradox)

| Analysis | AOR for "Always vs. Rarely" handwashing | 95% CI | p-value |
| --- | --- | --- | --- |
| Full model (adjusted for interviewer type) | 0.63 | 0.45–0.87 | 0.005 |
| Restricted to external researcher interviews only (n=456) | 0.52 | 0.38–0.71 | <0.001 |
| Restricted to local health worker interviews only (n=744) | 1.18 | 0.87–1.60 | 0.29 |

Conclusion: Social desirability bias is present when interviews are conducted by local health workers. External researchers yield more valid self-reported behavioral data in this context. All behavioral variables (handwashing, nail trimming, vegetable washing) should be interpreted with this limitation in mind.

# Table S8G: Sensitivity Analysis for Deworming History

| Analysis | AOR for HDS High vs. None | 95% CI | p-value |
| --- | --- | --- | --- |
| Full model (adjusted for deworming) | 2.89 | 2.01–4.15 | <0.001 |
| Excluding children who received deworming in past 6 months (n=788) | 2.94 | 2.11–4.09 | <0.001 |
| Restricted to children who received deworming (n=412) | 2.67 | 1.89–3.77 | <0.001 |

Interpretation: The HDS–IPI association remained strong and significant regardless of deworming history, confirming that the graded association is not an artifact of differential deworming coverage.
