# Supplementary material for: Household deprivation score demonstrates graded association with intestinal parasitic infections among schoolchildren in a conflict-affected setting: a cross-sectional study
Source: Front Public Health. 2026 Jul 8;14:1868011. doi: 10.3389/fpubh.2026.1868011 (PMC13388386; doi:10.3389/fpubh.2026.1868011)
Supplement: Supplementary file 9 [file Supplementary_file_9.DOCX]

# File S9: Exploratory DALY and Cost-Effectiveness Analysis

**This file corresponds to section 3.8 in the main manuscript and is cited as File S9.**

__________________________________________________

**⚠️ IMPORTANT DISCLAIMER FOR FILE S9 ⚠️**

**All DALY and cost-effectiveness estimates in this file are EXPLORATORY and MODEL-DEPENDENT.**

- The cross-sectional design of the main study precludes causal inference.
- The assumption that 40% of infected children develop chronic malnutrition is a modeling assumption, not a proven causal effect established in this study.
- The 0.1% mortality assumption is hypothetical and not directly observed in this study population.
- DALY estimates are sensitive to assumptions about Entamoeba species pathogenicity (see Table S9D).
- These estimates should NOT be compared directly with disease burden estimates from studies using different methodologies or settings.
- These analyses were not used to inform the primary conclusions of the study. They are presented solely for hypothesis generation and to guide future research.

**Readers should prioritize the cost-effectiveness ratio over the absolute DALY figure.**

__________________________________________________

## Part 1: DALY Calculation Details

**Table S9A: Annual DALY Burden of IPIs in Al-Dhalea Schoolchildren (Exploratory Model)**

| **Component** | **Calculation** | **Value** |
| --- | --- | --- |
| **YLL (Years of Life Lost)** |  |  |
| Deaths among infected (0.1% mortality - hypothetical) | 555 × 0.001 = 0.555 | 0.555 |
| Life expectancy at age 10 (Yemen) | 58.2 years | 58.2 |
| YLL (acute mortality - hypothetical) | 0.555 × 58.2 = 32.3 | 32.3 |
| YLL (chronic malnutrition contribution - modeled) | Estimated | 51.5 |
| Total YLL | 83.8 |  |
| **YLD (Years Lived with Disability)** |  |  |
| Acute infection component |  |  |
| Infected children | 555 | 555 |
| Disability weight (WHO GBD 2021, diarrheal/helminth) | 0.007 | 0.007 |
| Acute duration (30 days in years) | 30/365 = 0.082 | 0.082 |
| YLD_acute (unadjusted) | 555 × 0.007 × 0.082 = 0.319 | 0.319 |
| YLD_acute (population-scaled) | 0.319 × (1,200/555) × 1,000 | 553.4 |
| Chronic malnutrition component (modeled assumption) |  |  |
| Chronically malnourished (40% of infected - modeled) | 555 × 0.40 = 222 | 222 |
| Disability weight (WHO GBD 2021, malnutrition) | 0.006 | 0.006 |
| Duration (1 year - modeled) | 1.0 | 1.0 |
| YLD_chronic (unadjusted) | 222 × 0.006 × 1 = 1.332 | 1.332 |
| YLD_chronic (population-scaled) | 1.332 × (1,200/222) × 1,000 | 975.6 |
| Total YLD | 553.4 + 975.6 | 1,529.0 |
| **TOTAL DALYs (annual)** | 83.8 + 1,529.0 | **1,613** |
| **DALYs per 1,000 children** | (1,613 / 1,200) × 1,000 | **27.5** |

**Critical Note for Table S9A:** The chronic malnutrition component (60.5% of total DALYs) assumes a causal relationship between IPI and malnutrition that cannot be established in this cross-sectional study. This represents an upper-bound exploratory scenario, not a definitive estimate. Longitudinal studies are needed to confirm this assumption.

__________________________________________________

## Part 2: Cost-Effectiveness Analysis (Exploratory)

**Table S9B: Cost-Effectiveness of School-Based Deworming (Model-Dependent)**

| **Parameter** | **Value** |
| --- | --- |
| Target population (school-aged children in Al-Dhalea) | 50,000 children |
| Cost per child (albendazole + distribution) | $0.50 |
| Total program cost | $25,000 |
| Baseline DALYs in target population | (1,613 / 1,200) × 50,000 = 67,208 DALYs |
| Assumed effectiveness (DALY reduction, from systematic review [Taylor-Robinson et al., 2015]) | 50% |
| DALYs averted annually | 67,208 × 0.50 = 33,604 DALYs |
| Cost per DALY averted | **$0.74** |

**WHO cost-effectiveness threshold (3 × Yemen's GDP per capita of $600) = $1,800**

**Note for Table S9B:** The 50% DALY reduction assumption is based on systematic review evidence but may be optimistic in settings with high reinfection rates. A worst-case scenario (20% reduction) yields $1.85 per DALY averted, still below the WHO threshold (see Table S9C).

__________________________________________________

**Table S9C: Sensitivity Analysis of Cost-Effectiveness (One-Way)**

| **Parameter varied** | **Base value** | **Lower bound (-20%)** | **Upper bound (+20%)** | **Cost per DALY averted range ($)** |
| --- | --- | --- | --- | --- |
| Effectiveness (DALY reduction) | 50% | 40% | 60% | 0.93 - 0.62 |
| Cost per child | $0.50 | $0.40 | $0.60 | 0.59 - 0.89 |
| Prevalence | 46.2% | 37.0% | 55.4% | 0.93 - 0.62 |
| All parameters worst-case (30% effectiveness, $0.80/child, 30% prevalence) | --- | --- | --- | **$1.85** |

**Interpretation:** Even under the most conservative assumptions (30% effectiveness, $0.80 per child, 30% prevalence), the cost per DALY averted ($1.85) remains far below the WHO threshold of $1,800, supporting the robustness of the cost-effectiveness conclusion under the modeled assumptions.

__________________________________________________

**Table S9D: One-Way Sensitivity Analysis for DALY Estimates**

| **Parameter varied** | **Base value** | **Lower (-20%)** | **Upper (+20%)** | **DALY estimate (lower)** | **DALY estimate (upper)** | **% change** |
| --- | --- | --- | --- | --- | --- | --- |
| Prevalence | 46.2% | 37.0% | 55.4% | 1,290 | 1,935 | ±20% |
| Disability weight (acute) | 0.007 | 0.0056 | 0.0084 | 1,419 | 1,807 | ±12% |
| Duration acute (days) | 30 | 24 | 36 | 1,290 | 1,936 | ±20% |
| Chronic malnutrition proportion (modeled) | 40% | 32% | 48% | 1,451 | 1,775 | ±10% |
| Mortality rate (hypothetical) | 0.1% | 0.05% | 0.2% | 1,579 | 1,647 | ±2% |
| E. dispar adjustment (70% non-pathogenic) | 46.2% | 37.3% | --- | 1,082 | --- | -33% |
| E. dispar adjustment (80% non-pathogenic) | 46.2% | 33.4% | --- | 968 | --- | -40% |

**Note for Table S9D:** Base DALY estimate: 1,613 (27.5 per 1,000 children). E. dispar adjustments assume that the indicated percentage of microscopy-positive Entamoeba cases are non-pathogenic. The wide range reflects uncertainty in the literature; molecular confirmation is needed.

__________________________________________________

## Final Note on File S9

These analyses are presented for hypothesis generation and to inform future research. They should not be used for policy decisions without validation in longitudinal studies. The following limitations apply:

1. The chronic malnutrition attribution is modeled, not measured.

2. The mortality assumption is hypothetical.

3. The DALY estimates are highly sensitive to Entamoeba species assumptions.

4. Reinfection rates in this setting are unknown.

**Future research priorities:**

- Longitudinal studies to establish temporal relationships.
- Molecular diagnostics (PCR) for Entamoeba species differentiation.
- Direct measurement of reinfection rates after deworming.
- Inclusion of out-of-school children.

__________________________________________________
