# Supplementary material for: Household deprivation score demonstrates graded association with intestinal parasitic infections among schoolchildren in a conflict-affected setting: a cross-sectional study
Source: Front Public Health. 2026 Jul 8;14:1868011. doi: 10.3389/fpubh.2026.1868011 (PMC13388386; doi:10.3389/fpubh.2026.1868011)
Supplement: Supplementary file 10 [file Supplementary_file_10.DOCX]

File S10: HDS Screening Tool (Field Use)

Household Deprivation Score (HDS) for IPI Risk Stratification

For use by community health workers in conflict-affected and resource-limited settings

# Instructions:

Ask the parent/guardian these three questions. For each "YES" answer, add 1 point.

## ❓ QUESTION 1: URBAN POVERTY

"Is your household poor AND do you live in an urban area (city/town)?"

YES (+1 point) – May indicate overcrowding, poor sanitation, limited healthcare access in informal settlements
NO (0 points)

## ❓ QUESTION 2: POVERTY

"Is your household poor (unable to afford basic needs like food, soap, or healthcare)?"

YES (+1 point) – Economic constraints limit access to protective measures
NO (0 points)

## ❓ QUESTION 3: RURAL POVERTY

"Is your household poor AND do you live in a rural area (village/remote area)?"

YES (+1 point) – Geographic marginalization, reduced access to services
NO (0 points)

# 📊 TOTAL HDS SCORE

Sum all points (0–3)

| HDS Score | Category | Risk Level | Recommended Action |
| --- | --- | --- | --- |
| 0 | None | Low | Routine health education |
| 1 | Low | Medium | Health education + monitor |
| 2 | Medium | High | Prioritize deworming + nutrition support |
| 3 | High | Very High | Immediate deworming + nutrition support + hygiene kit |

## 📈 Evidence from our study (N = 1,200)

| HDS Category | IPI Prevalence | Adjusted Odds Ratio (95% CI) |
| --- | --- | --- |
| None (0) | 31.4% | Reference |
| Low (1) | 42.2% | 1.45 (1.08–1.95) |
| Medium (2) | 53.3% | 2.12 (1.58–2.85) |
| High (3) | 71.2% | 2.89 (2.01–4.15) |

Footnote 1: IPI prevalence is the proportion of children in each HDS category who tested positive for at least one intestinal parasite.

Footnote 2: Adjusted Odds Ratio (AOR) from multilevel mixed-effects logistic regression, adjusted for age, sex, family size, parental education, water source, toilet type, handwashing practices, nail trimming, raw vegetable washing, animals in home, abdominal pain, diarrhea, BMI category, anemia status, deworming history, and interviewer type.

Footnote 3: All associations are statistical correlations from a cross-sectional study. Causal inference is not possible.

Children with HDS ≥ 2 have 2–3 times higher odds of intestinal parasitic infection (statistical association, not causal).

## ✅ Action Checklist for HDS ≥ 2:

- Administer deworming medication (albendazole 400 mg single dose)
- Provide iron supplementation if anemic
- Deliver hygiene kit (soap, nail clippers, water container)
- Educate family on: Handwashing with soap, Regular nail trimming, Animal confinement
- Schedule follow-up in 3 months

Developed from research in Al-Dhalea Governorate, Yemen (2025)

Contact: n.taleb@ust.edu | ORCID: 0000-0002-2266-1569

This tool is free to use and adapt for humanitarian purposes.

## ⚠️ IMPORTANT LIMITATION

This tool is for risk stratification and prioritization only, NOT for diagnosis.

- A positive screen (HDS ≥ 2) does NOT guarantee that a child has an intestinal parasitic infection.
- A negative screen (HDS = 0) does NOT rule out infection.
- All children with symptoms (abdominal pain, diarrhea, poor growth, visible worms in stool) should receive diagnostic testing regardless of HDS score.
- This tool is intended to help community health workers allocate limited resources (deworming medication, hygiene kits, nutrition support) to the most vulnerable households in humanitarian settings.
